# The molecular dimension of microbial species: 1. Ecological distinctions among, and homogeneity within, putative ecotypes of *Synechococcus* inhabiting the cyanobacterial mat of Mushroom Spring, Yellowstone National Park

**DOI:** 10.3389/fmicb.2015.00590

**Published:** 2015-06-22

**Authors:** Eric D. Becraft, Jason M. Wood, Douglas B. Rusch, Michael Kühl, Sheila I. Jensen, Donald A. Bryant, David W. Roberts, Frederick M. Cohan, David M. Ward

**Affiliations:** ^1^Department of Land Resources and Environmental Sciences, Montana State University, Bozeman, MTUSA; ^2^Single Cell Genomics Center, Bigelow Laboratory for Ocean Sciences, East Boothbay, MEUSA; ^3^J. Craig Venter Institute, Rockville, MDUSA; ^4^Marine Biological Section, Department of Biology, University of Copenhagen, HelsingørDenmark; ^5^Plant Functional Biology and Climate Change Cluster, University of Technology Sydney, Ultimo, NSWAustralia; ^6^The Novo Nordisk Foundation Center for Biosustainability, Technical University of Denmark, HellerupDenmark; ^7^Department of Biochemistry and Molecular Biology, The Pennsylvania State University, University Park, PAUSA; ^8^Department of Chemistry and Biochemistry, Montana State University, Bozeman, MTUSA; ^9^Department of Ecology, Montana State University, Bozeman, MTUSA; ^10^Department of Biology, Wesleyan University, Middletown, CTUSA

**Keywords:** Mushroom Spring, microbial species, microbial ecology, population genetics, thermophilic *Synechococcus*

## Abstract

Based on the Stable Ecotype Model, evolution leads to the divergence of ecologically distinct populations (e.g., with different niches and/or behaviors) of ecologically interchangeable membership. In this study, pyrosequencing was used to provide deep sequence coverage of *Synechococcus psaA* genes and transcripts over a large number of habitat types in the Mushroom Spring microbial mat. Putative ecological species [putative ecotypes (PEs)], which were predicted by an evolutionary simulation based on the Stable Ecotype Model (Ecotype Simulation), exhibited distinct distributions relative to temperature-defined positions in the effluent channel and vertical position in the upper 1 mm-thick mat layer. Importantly, in most cases variants predicted to belong to the same PE formed unique clusters relative to temperature and depth in the mat in canonical correspondence analysis, supporting the hypothesis that while the PEs are ecologically distinct, the members of each ecotype are ecologically homogeneous. PEs responded differently to experimental perturbations of temperature and light, but the genetic variation within each PE was maintained as the relative abundances of PEs changed, further indicating that each population responded as a set of ecologically interchangeable individuals. Compared to PEs that predominate deeper within the mat photic zone, the timing of transcript abundances for selected genes differed for PEs that predominate in microenvironments closer to upper surface of the mat with spatiotemporal differences in light and O_2_ concentration. All of these findings are consistent with the hypotheses that *Synechococcus* species in hot spring mats are sets of ecologically interchangeable individuals that are differently adapted, that these adaptations control their distributions, and that the resulting distributions constrain the activities of the species in space and time.

## Introduction

Across a great diversity of microbial habitats, closely related populations have frequently shown distinct distributions along environmental gradients (West and Scanlan, 1999; Béjà et al., 2001; Ward and Cohan, 2005; Johnson et al., 2006; Walk et al., 2007; Hunt et al., 2008; Manning et al., 2008; Lau et al., 2009; Martiny et al., 2009; Miller et al., 2009; Connor et al., 2010; Denef et al., 2010; Shapiro et al., 2012; Kashtan et al., 2014). These patterns have prompted the hypothesis that some microorganisms might exist as ecological species occupying distinct niches (Cohan, 1994; Ward, 1998; Ward and Cohan, 2005; Smith et al., 2006; Sikorski, 2008; Vos, 2011), as theorized in the Stable Ecotype Model of species and speciation (Cohan and Perry, 2007). However, there are two fundamental unresolved issues with these kinds of studies. First, it is unclear whether the molecular variation that has been observed is sufficient to resolve populations at the species level, especially in studies based on variation in highly conserved molecular markers, such as 16S rRNA. Second, most studies address only the first of two expectations of the Stable Ecotype Model, that ecological species be distinct from one another and thus able to coexist indefinitely. The model also has the expectation that the membership within each species must be ecologically homogeneous, so that lineages within a species cannot coexist indefinitely (Koeppel et al., 2013; Kopac et al., 2014). This second quality has presented a challenge to microbial ecology because it requires sufficient phylogenetic resolution to detect individuals within the most newly divergent, ecologically distinct populations. Until recently, investigating the ecological interchangeability of individuals within a naturally occurring population has not been a priority of microbial speciology (Kopac et al., 2014).

This is the first of a three-paper series concerned with the degree of molecular resolution needed to observe microbial species. Here we report high-resolution, theory-based molecular identification of *Synechococcus* species in a well-studied hot spring microbial mat community, and we test the ecological distinctions of these species by investigating their distributions, responses to perturbation, and differences in gene expression. Our observations have led to the prediction that there are *Synechococcus* species with distinct light adaptations. Our second paper reports on the cultivation of strains representative of some of these species and demonstrates their adaptations (Nowack et al., 2015, this issue). The third paper compares the genomes of these strains, which have begun to demonstrate possible mechanisms underlying their light adaptations, as well as unsuspected adaptations to other environmental parameters Olsen et al. (2015, this issue). As will be shown, these strains exhibited identical or nearly identical 16S rRNA gene sequences.

Earlier molecular studies of microbial mat communities in alkaline siliceous hot springs (Mushroom Spring and Octopus Spring, Yellowstone National Park, WY, USA) were limited by the molecular resolution of slowly evolving sequences. 16S rRNA gene analyses revealed several cyanobacterial (*Synechococcus*) variants identified as A′′, A′, A, B′ and B, which exhibited different distribution patterns between 72–74°C and ~50°C along the effluent channel flow path (Ferris and Ward, 1997), or in vertical position within the photic zone of the mat (Ramsing et al., 2000). Others have found similar results using 16S rRNA variation to study hot spring cyanobacterial distributions (Jing et al., 2006; Lau et al., 2009; Miller et al., 2009). However, subsequent studies of Mushroom Spring and Octopus Spring mats employing the more rapidly evolving 16S–23S rRNA internal transcribed region revealed the existence of ecologically distinct populations with the same 16S rRNA sequence and prompted concerns about the possible need for yet higher molecular resolution to detect ecological species populations (Ferris et al., 2003). Indeed, population diversity analyses (Melendrez et al., 2011) and fine-scale distribution analyses of *Synechococcus* sequence diversity along effluent flow and vertical gradients (Becraft et al., 2011) using protein-encoding loci showed that molecular resolution influences the identification of ecological species populations in the Mushroom Spring mat.

In these most recent studies, we used an evolutionary simulation algorithm based on the Stable Ecotype Model of speciation to predict ecological species, rather than using an arbitrary molecular divergence cutoff. The algorithm Ecotype Simulation (Koeppel et al., 2008) hypothesizes from neutral single-gene or multi-locus sequence variation which variants should be grouped into different ecological species, each of which represents a set of ecologically interchangeable individuals. Each of these populations is considered a putative ecotype (PE), because the output of the algorithm is a set of hypothesized ecological species, whose predicted properties of ecological distinctness among and interchangeability within groups have not yet been demonstrated. Ecotype Simulation models the sequence diversity within a phylogenetic lineage as the evolutionary result of net ecotype formation, periodic selection (Koch, 1974), and drift, yielding the number of PEs within an environmental sample and demarcating sequence variants grouped into each PE.

In Becraft et al. (2011), genetic variation at the *psaA* locus (encoding a major Photosystem I reaction center protein subunit) was analyzed because (i) it offers 7–10 times more molecular resolution than the 16S rRNA locus, (ii) it is known to be highly expressed *in situ* (Liu et al., 2012), (iii) it exists as a single copy in the genomes of mat *Synechococcus* isolates (Bhaya et al., 2007), (iv) the protein subunit it encodes (PsaA) is essential for photosynthesis, (v) it exhibits no evidence of recombination in the region studied and (vi) its structure is known (Jordan et al., 2001). While this gene might underlie some ecological distinctions among PEs, *psaA* is for our present purposes like any gene in the genomes of the populations being investigated. That is, the individuals within PEs are expected to accumulate neutral sequence divergence *in every gene* (including *psaA*), regardless of what physiological properties make PEs ecologically distinct. Thus, sequence clustering in any gene is expected to reveal ecotypes. To the extent that there is also adaptive divergence in *psaA*, this could supply additional resolution for distinguishing newly divergent ecotypes.

In Becraft et al. (2011), partial *psaA* sequences obtained by PCR amplification, cloning, and sequencing from samples collected along the effluent flow channel were used to demarcate *Synechococcus* PEs. The 320 cloned sequences analyzed suggested that each PE contained a dominant variant sequence that could, in most cases, be separated by denaturing gradient gel electrophoresis (DGGE) to demonstrate the distinctness of PE distributions along flow and vertical gradients. However, co-migration of DGGE bands made band purification and sequencing difficult, and without band sequences it was impossible to know whether different bands corresponded to the same or different PEs. Furthermore, the low number of sequences (320) and habitats sampled (25) limited the ability to identify different variants within PEs, making it impossible to examine the ecological interchangeability of individuals within a PE.

In this study we used pyrosequencing of PCR-amplified *psaA* genes and transcripts, which resulted in a more complete (>500 times greater coverage than in Becraft et al., 2011), sequence-based view of the genetic and ecological diversity within the *Synechococcus* populations in Mushroom Spring microbial mats. Pyrosequencing can overestimate the diversity within microbial populations due to a high rate of sequencing errors (Reeder and Knight, 2009), but because reference clone library sequences and genomes of representative *Synechococcus* isolates were available (Allewalt et al., 2006; Bhaya et al., 2007; Becraft et al., 2011), it was possible to recognize such artifacts by alignment to known reading frames. To test the ecological distinctions of PEs we (i) extended fine-scale distribution studies to include four temperatures and up to 12 depth intervals at multiple temperatures, (ii) investigated the responses of *Synechococcus* populations to perturbations of temperature and irradiance, and (iii) examined PE-specific transcription patterns over a diel cycle. Additionally, because the greater depth of coverage led to recovery of multiple sequence variants within PEs, it was possible to test for ecological homogeneity within PEs, based on the expectation that all members of an ecological species population would be co-distributed along environmental gradients and change uniformly in response to environmental perturbations. We provide evidence that most of the abundant PEs predicted by Ecotype Simulation are ecologically distinct from one another, and that the individuals of a given PE are ecologically homogeneous.

## Materials and Methods

### Sampling

This study focused on the mat community inhabiting the ~60–68°C region of the major effluent channel of Mushroom Spring, an alkaline siliceous hot spring in the Lower Geyser Basin, Yellowstone National Park, WY, USA. Samples were collected from sites spread over a distance of ~10 m along the main flow path and were defined by temperatures measured at the time of collection (see Becraft et al., 2011 Supplementary Information). Mat samples for distribution studies were collected in duplicate on 12, 13, 14, and 15 September 2008 (60, 63, and 65°C), and 12 and 13 September 2009 (68°C) using a #2 cork borer (19.6 mm^2^) and were immediately frozen in liquid N_2_, or, in the case of cores for vertical analysis, in isopentane cooled with liquid N_2_ to minimize decomposition of nucleic acids and to preserve core integrity (Ramsing et al., 2000). The latter cores were subsequently dissected at ~80 μm intervals using a cryotome, as described in Becraft et al. (2011). Samples for transcript analysis over a diel cycle were taken at hourly intervals starting at 1700 h on 11 September 2009, and continuing until 1600 h on 12 September 2009 using a #4 cork borer (38.5 mm^2^). Duplicate samples were collected from within a ~1 m^2^ area at a 60°C site and pooled.

### Perturbation Experiments

#### Temperature Shift

Samples for temperature-shift experiments were collected in duplicate using a #2 cork borer, placed in their original vertical orientation into 3-ml glass vials that were filled with spring water, capped, and suspended in the effluent channel at a higher temperature site by aluminum wires attached to wooden stakes on either side of the channel (see Supplementary Figure S1A). Duplicate samples were retrieved 2 and 4 days after the disturbance was initiated on 27 October 2008. Previous studies showed that confining samples in this way did not alter the initial population structure of samples when incubated at the collection site (i.e., not shifted in temperature; Ruff-Roberts et al., 1994). Some bleaching was noticed in later stages of the incubation period, likely due to the lack of flow in the closed vials, which severely impacts diffusion, or possibly to other inhibiting factors, such as the accumulation of toxic metabolites.

#### Light Alteration

On 27 October 2008, a 254 cm^2^ rectangular wooden frame covered with four layers of stretched muslin (Supplementary Figure S1B) was placed ~2.5 cm above the mat surface at a ~63°C site to reduce ambient solar irradiance by ~92%. Duplicate samples were retrieved 2 and 4 days after the disturbance was initiated on 27 October 2008. All samples were immediately frozen on dry ice (-78.5°C) in the field and kept frozen at -80°C until analysis.

#### Molecular Methods

DNA was extracted and purified as described in Becraft et al. (2011), and RNA was extracted and purified as described in Liu et al. (2011). Primers for the amplification of *Synechococcus* A/B-lineage *psaA* genes (psaAcenterforward: 5′-TTCCACTACCACAAGCGGGCTCC-3′, psaAreverse: 5′-CAGGCCACCCTTGAAGGTG-3′) were designed to yield a 324 bp segment to maximize the number of single-nucleotide polymorphisms (SNPs) that could be used to differentiate PEs, and were tested for specificity to A/B′-like *Synechococcus* sequences as described in Becraft et al. (2011). The SNPs in this region of the *psaA* gene are unlikely to be under positive selection, as described in Supplementary Information Section I. The reduced sequence length in this study eliminated nucleotide diversity in some cases. This caused sequences representative of subclades PE A1-3 and A1-4, identified in Becraft et al. (2011) on the basis of 523-nt sequences, to be combined into a single high-frequency sequence variant (PE A1 in this study). Also, sequences representative of subclade PE A′9-2 were combined with those of PE A′9 [previously labeled A9 in Becraft et al. (2011); see **Table 1**, which compares A-like and A′-like PEs from this and the previous study]. Thus, it is possible that PEs A1 and A′9 each include > 1 ecotype and that the sequence analyzed is too conserved to identify these populations accurately.

**Table 1:** Summary of non-singleton fine-scale demarcated A and A′-like putative ecotype (PE) average population percentages for individual habitat samples corresponding to distributions, expressing timing and responses to environmental perturbations.

			Percent population and vertical orientation^2^												Perturbation response^3^	
			2006 cloning & sequencing					2008/09 Ti454 bar code								
PE Ti454	# CCA clusters	Becraft et al. (2011) designation^1^	60°C	V	63°C	V	65°C	60°C	V	63°C	V	65°C	V	68°C	60 →65°C	Light reduction
A1	1	A1 (A1-3, A1-4)	9.1	↓	43.8	↕	6.2	2.6	↓	2.9	↓	26.8	↕	4.7	↑	↑
A2		A2	0.4		0.0		3.3	0.2		0.3		0.4		2.0	–	↓
A3		A3	1.1		0.0		0.0	0.1		0.1		0.5		0.0	–	–
A4	1	A4	4.2		0.0		0.0	2.7	↓	0.5		3.6	↓	0.6	↑	↓
A5		A5	1.5		0.0		0.0	1.5		1.0		8.1	↓	0.6	↑	↑
A6	1	A6	3.8		5.6		1.6	6.4	↓	3.2	↓	13.2	↔	1.9	↓	↓
A7	1	A7	1.1		50.0	↑	1.6	0.0		0.2		4.5	↕	3.2	–	↑
A′8		A′8	0.0		0.0		18.0	0.0		0.0		0.0		10.7	–	–
A′9		A′9 (A′9-2)	0.0		0.0		52.5	0.0		0.0		0.3		23.1	–	–
A10								0.3		0.2		0.8		0.5	–	↓
A11								0.1		0.1		1.4		0.1	–	↓
A12	1	A1-2	0.0		16.7		0.0	0.1		0.3		12.6	↕	1.8	–	↑
A13								0.0		0.0		0.1		0.2	–	–
A14	1	A1-1	3.8		0.0		0.0	2.0	↓	1.3	↓	10.7	↔	0.6	↑	↑
A15								0.1		0.0		0.7		0.1	↑	–
A′16								0.0		0.1		0.2		8.3	–	–
A′17		A′9-1	0.0		0.0		11.5	0.0		0.0		0.0		14.6	–	–
A′18								0.0		0.0		1.2		0.4	–	–

*Putative ecotypes without identified vertical distributions or perturbation responses (arrows) were too low in relative abundance to track with confidence. Highlighted rows correspond to predominant PEs discussed in the main text.*

*^1^Putative ecotype subclades that were demarcated as a single ecotype in Becraft et al. (2011) that were separately demarcated in barcode analyses are indicated with hyphenated numbers next to PE designations. PE subclades that were not detected in barcode analysis due to decreased sequence length are indicated in parentheses next to the PE they were demarcated with in Becraft et al. (2011).*

*^2^‘V’ indicates vertical positioning at a specified temperature. Arrows under ‘V’ indicate highest relative abundance in upper green mat layer (↑), highest relative abundance in lower portion of the upper green mat layer (↓), highest relative abundance in center layers (↔), and relative abundance through all layers (↕).*

*^3^Perturbation responses are indicated by ↑ for increase in relative abundance, ↓ for decrease, and – for no change.*

To estimate ecotype-specific expression patterns, environmental nucleic acids were treated with DNase, and *psaA* cDNA was synthesized from RNA using the psaAreverse primer (see above) with the SuperScript III First-Strand Synthesis Supermix (Invitrogen, Carlsbad, CA, USA) according to the instructions of the manufacturer, and the product was amplified by PCR according to protocols described in Becraft et al. (2011). Controls containing only RNA (i.e., no reverse transcription step) were used during amplification to insure that all DNA had been degraded. Barcoding and Ti454 sequencing were completed at the J. Craig Venter Institute according to the GS FLX Titanium Series Rapid Library Preparation Method Manual. DNA was sheared using the Covaris S2 System, and qPCR was used to estimate accurately the number of molecules needed for emulsion PCR^1^ Sequences were submitted to MG-RAST (4613896.3-4614007.3).

### Identification of Sequences Containing Homopolymeric Artifacts

The resulting sequence data were first processed to remove homopolymeric errors (base pair insertions or gaps following strings of identical nucleotides) and sequences of poor quality. To generate alignments, we employed a Perl script from the Pigeon package (homopolymer-extinguisher.pl^2^) that uses ClustalW (Larkin et al., 2007) to align each raw sequence (plus its complement, reverse, and reverse complement sequences) with an in-frame consensus reference sequence of all *psaA* sequences from Becraft et al. (2011). Nucleotides in the raw sequences that caused a gap to form in the reference sequence reading frame with the best alignment of these sequences were assumed to result from erroneous homopolymers (Quince et al., 2009, 2011; Reeder and Knight, 2009), and these nucleotides were excised. This step also trimmed the raw sequences to the same length as the reference sequence. Any trimmed raw sequences containing gaps relative to the consensus reference sequence were removed from further analysis. This yielded 164,467 sequences, with an average of 1,713 (SD of 251) for each of 96 unique environmental samples. Separately, 17 total cDNA samples yielded, on average, 360 sequences per sample (SD of 120). cDNA sequences were trimmed to a 247 bp segment to obtain the maximum number of sequences for analysis.

### Putative Ecotype Demarcation

It was computationally too challenging to analyze all sequence variation using the current version of Ecotype Simulation. Thus, a Perl script from the Pigeon package (hfs-finder.pl^2^) was used to generate a list of high-frequency sequences by counting the number of occurrences of each unique sequence. Unless otherwise specified, high-frequency sequences were defined as unique sequence types with >50 identical representatives across all samples (96 DNA and 17 cDNA), which included the dominant variants of each PE identified in Becraft et al. (2011). High-frequency sequences showed no evidence of recombination in the region studied using the RDP3 software package (Martin et al., 2010). Each high-frequency sequence was assigned as either *Synechococcus* A-like or B′-like if it was ≥95% identical to the respective *psaA* genomic homologs in *Synechococcus* strain A (JA-3-3Ab) or B′ (JA-2-3B′a (2-13)) (Bhaya et al., 2007). Because no genome sequence was available for the A′ lineage [as defined by 16S rRNA sequence variation (Ferris and Ward, 1997)], we classified *psaA* sequences to the A′ lineage if they showed the highest similarity to 68°C metagenomic library sequences, which contain predominantly A′-like variants (see Supplementary Information in Becraft et al., 2011; Klatt et al., 2011), and these were included in the A-like phylogeny. Separate analyses were performed on the pool of A′-like plus A-like high-frequency sequences and for B′-like high-frequency sequences using Ecotype Simulation (with 1.5x sorting) to predict the number of PEs (Cohan and Perry, 2007; Koeppel et al., 2008). Neighbor-joining trees were constructed and uploaded into Ecotype Simulation as Newick files for ecotype demarcation. Two methods for Ecotype Simulation ecotype demarcation have been developed (Becraft et al., 2011; Melendrez et al., 2011; Francisco et al., 2012; Kashtan et al., 2014), both of which were used. The more conservative approach tends to yield PEs that are more inclusive clades; here a PE is demarcated as the most inclusive phylogenetic group for which the confidence interval for the number of predicted ecotypes includes the value 1. In our alternative fine-scale demarcation, PEs are demarcated as the largest groups for which the maximum likelihood solution for the number of predicted PEs equals the value 1. The Ecotype Simulation software and instructions for its use are freely available online^3^

### Determination of All Variants within Putative Ecotype Populations

The set of high-frequency sequences was de-replicated (i.e., to include only one example of each sequence). Two neighbor-joining trees were created, one for all unique A-like plus A′-like sequences, and the other for B′-like sequences, using Phylip’s dnadist and neighbor-joining programs (Felsenstein, 1989). When a low-frequency sequence fell within a clade of high-frequency sequences that were demarcated to a single PE, it was classified to that PE. When a low-frequency sequence did not fall within a clade of high-frequency sequences, it was tentatively assigned to the most closely related PE comprised of high-frequency sequences. All high-frequency sequences (and associated low-frequency sequences) were combined to obtain the total number of variants detected for each PE in each sample (PE sequences = dominant variant sequence + all other high-frequency sequences + all low-frequency sequences). The percentage contribution of each PE in each environmental sample was calculated by dividing the total number of sequences within a PE detected in a sample by the total number of *psaA* sequences in that sample [(PE sequences in sample/all associated sequences in sample) × 100]. Absolute quantitation of each PE’s abundance could not be achieved in these analyses because dispersed SNP patterns prevented the development of SNP-specific primers or probes, and the PCR and sequencing method employed only sampled proportions of populations in the data.

### Gene Expression Analyses

Transcripts obtained in sequence analysis were aligned with *psaA* gene sequences, which had been demarcated into PEs, to determine the number of transcripts per PE in each sample (no mismatches were allowed). This was divided by the total number of *psaA* transcripts retrieved in a sample, and the results are expressed as percent relative abundance at each time point.

Diel metatranscriptomic datasets described by Liu et al. (2012) were analyzed by using the Burrows–Wheeler Aligner (BWA) to identify transcripts for specific photosynthesis and N_2_ fixation genes (see Liu et al., 2012 for genes analyzed) associated with the genomes of either *Synechococcus* A (JA-3-3Ab) or B′ (JA-2-3B′a (2-13)) (Bhaya et al., 2007). We used the methods described in Liu et al. (2011, 2012), except that we recruited transcripts using genomes, instead of metagenomic assemblies. Because the transcript sequences were only 50 nt in length, we allowed up to 5 SNP differences to capture transcripts that matched with the diverse variants of B′-like and A-like populations. Raw transcript counts were normalized by the total number of transcripts recruited by that genome at each time point and then by the geometric mean of normalized transcript counts for all time points (Liu et al., 2011, 2012).

### Canonical Correspondence Analysis

The distributions of high-frequency *psaA* sequence variants in vertical subsections of duplicate cores, which had been collected from 60, 63, and 65°C sites, were analyzed using canonical correspondence analysis (CCA; Ter Braak, 1986; Legendre and Legendre, 1998) from the Vegan R package (Oksanen et al., 2013). A custom version of ordtest from the labdsv R package (Roberts, 2013) was used to test each PE for nonrandom distribution, and a custom plotting function was written to display the data (CCA-plot.R, Supplemental Information). This permitted us to evaluate whether high-frequency sequence variants of the same PE showed restricted distributions in the ordination space, and whether high-frequency sequence variants of different PEs showed disjunct distributions. The variation in distribution among variants was analyzed with respect to temperature and depth as linear predictors.

### Statistical Analyses

*G*-tests were conducted on *psaA* sequence counts along all environmental gradients for abundant PEs, and for A-like and B′-like PEs separately, to determine whether there was statistical significance for heterogeneity of PE distributions (see Supplementary Table S1). Analysis of co-variance (ANCOVA) tests were conducted for perturbation experiments to determine whether PE populations differed in their response to environmental change (see Supplementary Table S2). *G*-tests and ANCOVA analyses were done in Stata (StataCorp LP, Stata Statistical Software: Release 13, College Station, TX, USA). Additionally, over the course of the light-reduction and temperature-shift experiments, we tested whether PEs changed their relative frequencies, and we tested whether the membership of high-frequency sequences (and associated low-frequency sequences) in a given PE changed in unison. This was done by evaluating whether the percentage of each high-frequency sequence within a PE, and the ratio of low-frequency sequences to high-frequency sequences within a PE, changed separately over time using a generalized linear model (a flexible generalization of ordinary linear regression that allows for binomial distributions; see Supplementary Table S3).

### Microsensor Measurements

Diel sampling for molecular analyses was closely coordinated with simultaneous logging of the incident downwelling solar irradiance and *in situ* microsensor measurements of O_2_ concentration profiles in the microbial mats at the sample site following the same calibration and measurement procedures described in detail in earlier studies (Becraft et al., 2011; Jensen et al., 2011). At the end of the diel measurements, ~3 cm^2^ of mat was sampled with a glass corer and transported to the laboratory in spring water for subsequent measurements (within 24 h) of spectral light penetration using fiber-optic scalar irradiance microsensors (see details in Kühl, 2005; Ward et al., 2006).

Scalar irradiance spectra measured at particular depths of the mat were normalized to the incident downwelling irradiance at the mat surface and expressed in percent of downwelling irradiance at the mat surface. Additionally, measured scalar irradiance spectra in each depth were integrated over 400–700 nm [i.e., photosynthetic active radiation (PAR)] and normalized to the downwelling irradiance of PAR at the mat surface. This depth profile of E_0_(PAR) (in percent of downwelling irradiance) was then used together with the actual measured downwelling photon irradiance at the field site during the diel study to construct depth profiles of *in situ* E_0_(PAR) in units of μmol photons m^-2^ s^-1^ for all sampled time points in the diel study.

Isopleth diagrams of O_2_ concentration and photon scalar irradiance, E_0_(PAR) depth distribution over the diel cycle were constructed from depth profiles measured at different time points using the graphing software Origin Pro 8.5 (Origin Lab Corp., Northampton, MA, USA).

## Results

### Ecotype Simulation Analysis and Sequence Variation within and Among Putative Ecotypes

**FIGURE 1:**
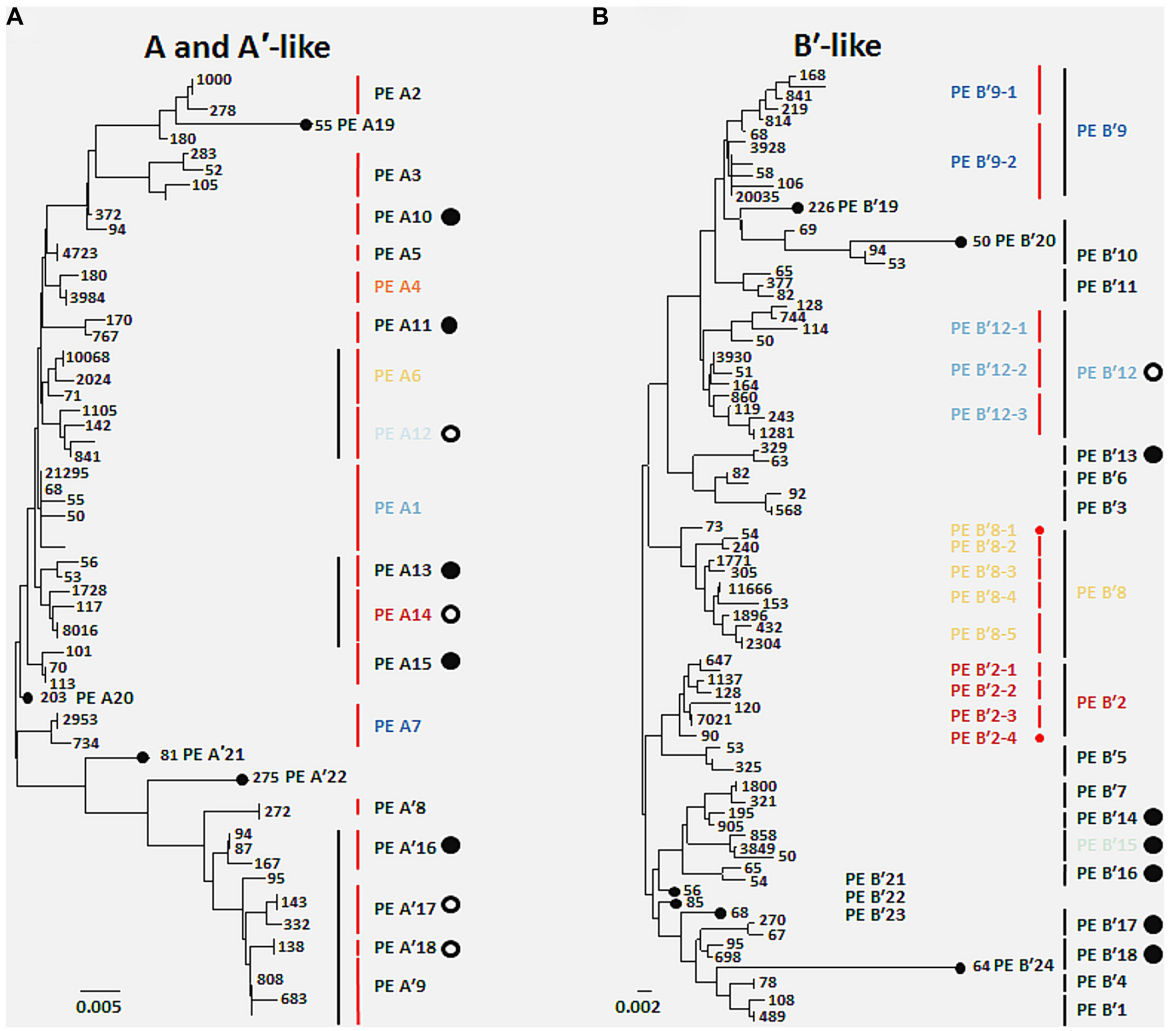
**Comparison of putative ecotypes (PEs) demarcated from high-frequency *psaA* sequences by Ecotype Simulation using the fine-scale and conservative demarcation approaches in the **(A)** A/A′ lineage and the **(B)** B′ lineage.** Black vertical bars indicate PEs demarcated using the conservative approach and red vertical bars indicate PEs demarcated using the fine-scale approach. Numbers indicate the total number of sequences sampled with a particular sequence across all 96 DNA samples. Large filled circles represent PEs newly identified in the present sequencing analysis, and large open circles indicate previously identified ecotype subclades in cloning and sequencing studies now demarcated as separate PEs. Small filled circles indicate PEs based on single high-frequency sequences. Colored PE designations represent predominant populations discussed in the main text, and colors correspond across all figures. Scale bars represent **(A)** 0.005 and **(B)** 0.002 substitutions per site.

A total of 119 unique high-frequency sequences were found in the variation detected in 113 environmental samples (96 DNA; 17 cDNA), and these sequences were analyzed using Ecotype Simulation to predict PEs (**Figure 1**). Ecotype Simulation analysis of high-frequency sequences from the clade of A-like and A′-like lineages predicted 22 PEs using fine-scale demarcation (15 and 7 PEs, respectively) and analysis of the B′-like lineage predicted 24 PEs in conservative demarcation analyses (**Tables 1** and **2**, and **Figure 1**). Results from the alternative demarcation approaches are also shown in **Figure 1**. The choice of demarcation approach was initially guided by PE distributions identified in Becraft et al. (2011) and high-frequency sequence distributions in the current study.

**Table 2:** Summary of non-singleton conservatively demarcated B′-like PE average population percentages for individual habitat samples corresponding to distributions, expression timing and responses to environmental perturbations.

			Percent population and vertical position^2^												Perturbation response^3^	
			{2006 cloning & sequencing					2008/09 Ti454 bar code								
PE Ti454	# CCA clusters	Becraft et al. (2011) designation^1^	60°C	V	63°C	V	65°C	60°C	V	63°C	V	65°C	V	68°C	60 →65°C	Light reduction
B′1		B′1	6.9		0.0		0.0	0.4		0.3		0.0		0.4	–	–
B′2*	1	B′2	3.1		0.0		0.0	15.7	↓	9.7		1.4		0.5	↓	–
B′3		B′3	2.3		0.0		0.0	0.1		0.3		0.0		0.9	–	–
B′4		B′4	4.2		0.0		0.0	0.0		0.2		0.0		0.0	–	–
B′5		B′5	1.1		0.0		0.0	0.0		0.3		0.1		0.0	–	–
B′6		B′6	1.1		0.0		0.0	0.1		0.0		0.0		0.8	–	–
B′7	1	B′7	8.0	↑	0.0		0.0	0.8		5.2	↑	0.1		0.2	–	–
B′8*	3	B′8	8.4		0.0		0.0	8.6	↑	2.5		1.6		1.0	↓	–
B′9*	1	B′9	29.9	↑	0.0	↑	0.0	23.5	↑	27.9	↑	3.4		3.2	↓	↓
none		B′10	1.1		0.0		0.0						
none		B′11	1.1		0.0		0.0						
none		B′12	0.8		0.0		0.0						
B′10								0.2		0.0		0.2		0.0	–	–
B′11								0.6		1.0		0.1		0.1	–	–
B′12*	2	B′9-1 and B′9-2	11.1		0.0		0.0	4.8	↑	7.8		1.0		1.1	↓	–
B′13								0.1		0.2		0.0		0.1	–	–
B′14								0.0		1.0		0.0		0.1	–	↑
B′15	1							0.2		0.3	↑	5.1		1.0	↑	–
B′16								0.0		0.4		0.0		0.0	–	–
B′17								0.9		0.8		0.0		0.0	–	–
B′18								0.8		0.7		0.1		0.0	–	–

*PEs without identified vertical distributions or perturbation responses (arrows) were too low in relative abundance to track with confidence. Highlighted rows correspond to predominant PEs in main-text.*

*^1^Putative ecotype subclades that were demarcated as a single ecotype in Becraft et al. (2011) that were separately demarcated in barcode analyses are indicated with hyphenated numbers next to PE designations.*

*^2^‘V’ indicates vertical positioning at a specified temperature. Arrows under ‘V’ indicate highest relative abundance in upper green mat layer (↑), highest relative abundance in lower portion of the upper green mat layer (↓), highest relative abundance in center layers (↔), and relative abundance through all layers (↕).*

*^3^Perturbation responses are indicated by ↑ for increase in relative abundance, ↓ for decrease, and – for no change.*

*^∗^B′-like PEs that contained one or more high frequency sequences that did not correspond with the most abundant high-frequency sequence distributions in CCA analysis, and could be representative of multiple PEs.*

Many PEs predicted from sequences obtained by pyrosequencing (2008 collections) corresponded to PEs previously demarcated in cloning and sequencing studies (2006 collections; Becraft et al., 2011) and this correspondence is shown in **Tables 1** and **2**. All 22 of the newly discovered PEs were either based on a single sequence or were in low frequency (ranging from 0.05 to 3.8% of total sequences; average 0.8% ± 0.14 SE). Among these only PE B′15 had a frequency greater than 1% of the total sequences (Supplementary Tables S4–S13). Newly discovered PEs in pyrosequencing analyses were labeled sequentially following the PEs previously identified and numbered in Becraft et al. (2011). Ecotype Simulation calculated the following rates of periodic selection (sigma) and ecotype formation (omega): 2.71 (sigma) and 0.94 (omega) for the A-like plus A′-like sequences, and 1.86 and 0.94 for B′-like sequences, parameterized as the number of events (periodic selection or ecotype formation) per nucleotide substitution in the 324 bp of the *psaA* gene. These results indicated that the rate of periodic selection was higher than the ecotype formation rate.

Each high-frequency sequence within a PE was associated with a set of low-frequency sequences (<50 identical sequences in the entire database), which typically exhibited randomly distributed differences in SNPs (examples are shown in Supplementary Figure S3). The number of unique high-frequency sequences in each well-sampled PE (i.e., average of 1713 total sequences) was lower in A-like PEs (range 1–4; average 2.67 ± 0.42 SE) than in B′-like PEs (range 3–11; average 7.8 ± 1.46 SE; *P* = 0.0052 for a two-tailed *t*-test). Because we classified many high-frequency sequences per PE, we had an opportunity to test whether high-frequency variants predicted to be members of a PE were ecologically interchangeable. For clarity, in-text figures focus on predominant PEs defined as those that were >5% of the population in any sample and well-represented in the barcode sampling (i.e., >3000 sequences). This includes PEs that were unresolved in Becraft et al. (2011; shaded in **Tables 1** and **2**); results for all PE populations are presented in Supplementary Tables S4–S13.

### Distribution Along the Effluent Flow Path and with Depth within the Upper Green Mat Layer

**FIGURE 2:**
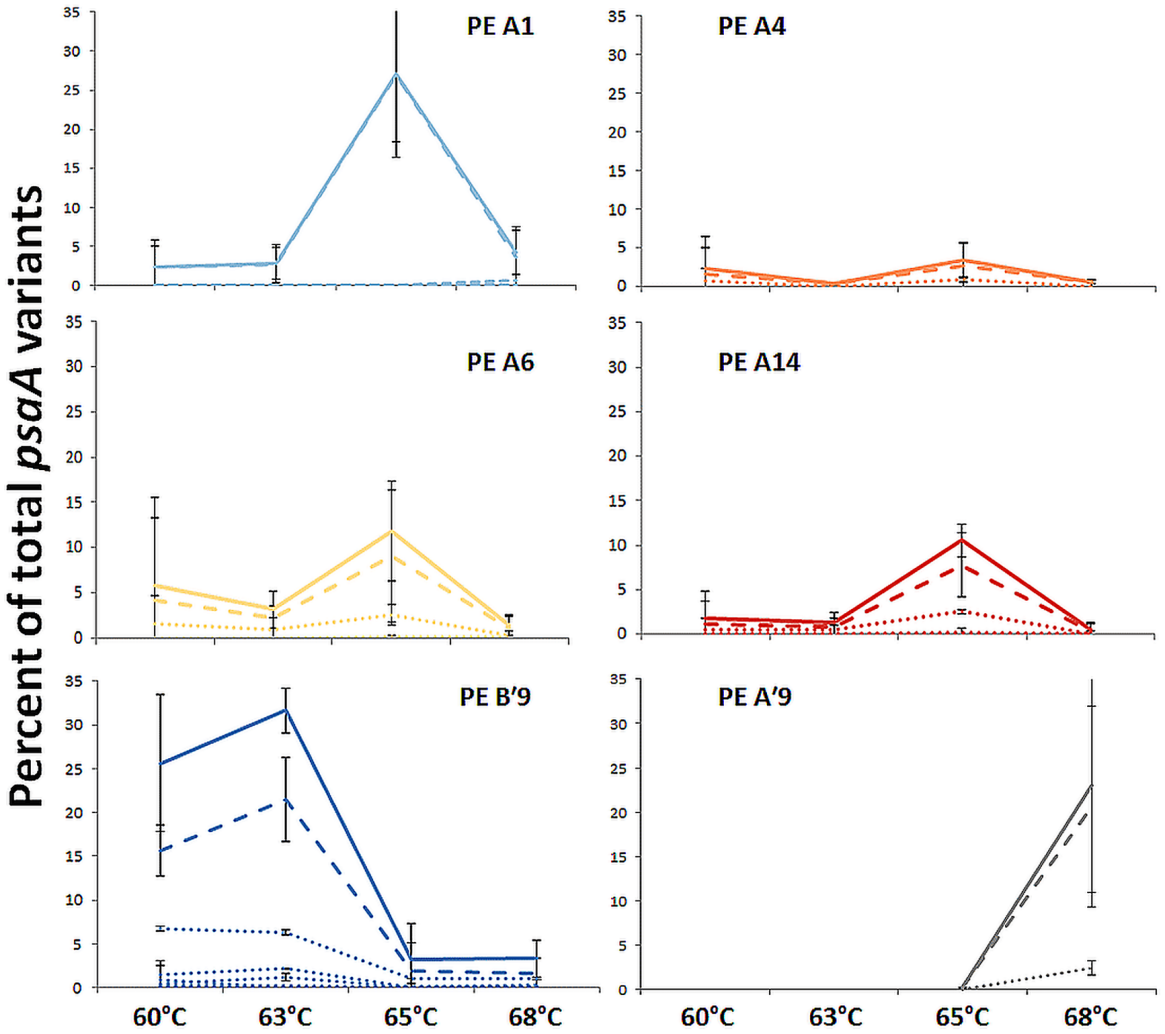
**Relative abundance of predominant PEs along the effluent flow path from 60 to 68°C.** Solid line represents all members of a PE, the dashed line represents the percentage of dominant variant sequences, and dotted lines indicate the percentage of each additional high frequency sequence variant. Bars indicate range of replicate samples (*n* = 2). Temperature distributions of other PEs are shown in **Tables 1 and 2**

**FIGURE 3:**
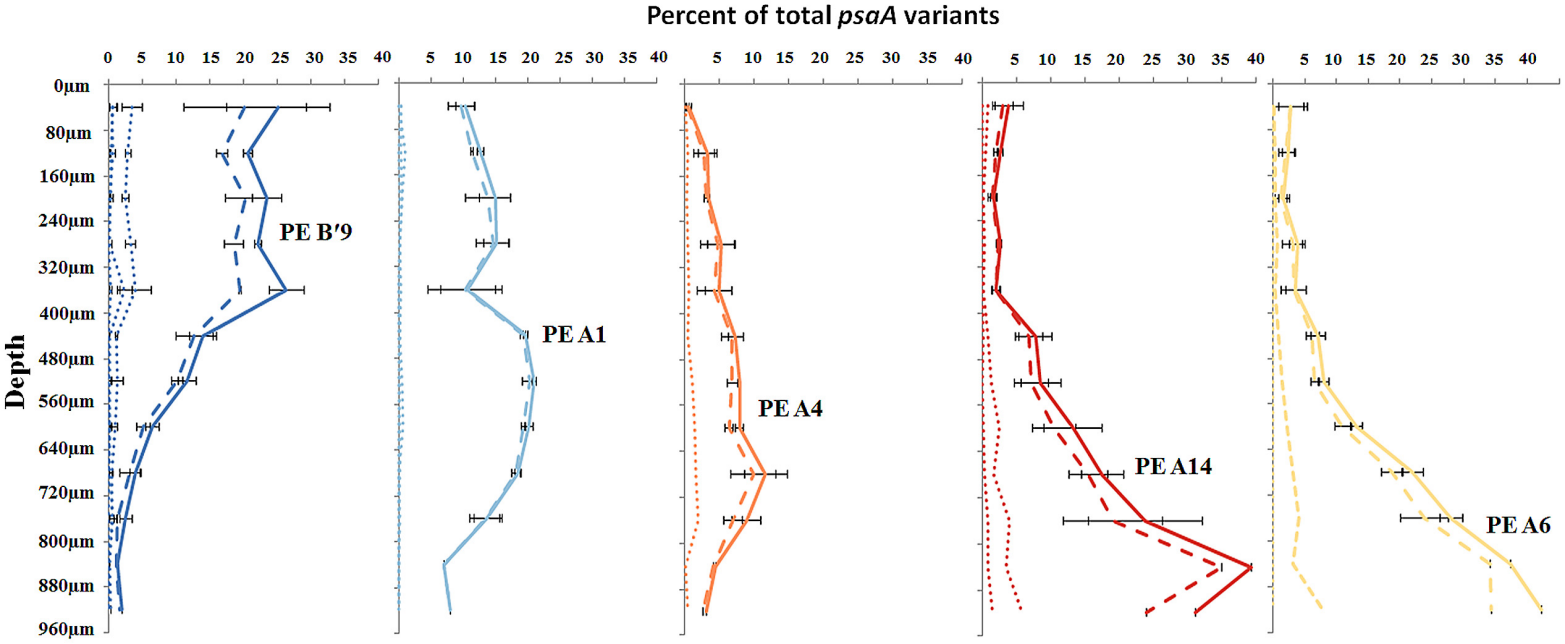
**Relative abundance of predominant putative ecotypes in ~80 μm subsections along the vertical gradient in 60–63°C mat samples.** Solid line represents all members of a PE, the dashed line represents the percentage of dominant variant sequences, and dotted lines indicate the percentage of each additional high frequency sequence variant. Bars indicate range of replicate samples (*n* = 2).

Most predominant PEs exhibited strong and largely unique associations with environmental gradients. For instance, PEs from the A′-like, A-like and B′-like were clearly distributed to high, middle and low temperature sites, respectively (**Figure 2**). At a given temperature, predominant PEs were also distributed differently with respect to depth in the upper green layer of the mat. For instance, at 60–63°C we observed a stratification of PE B′9 above PE A1 above PE A4 above PEs A6 and A14 (**Figure 3**).

**FIGURE 4:**
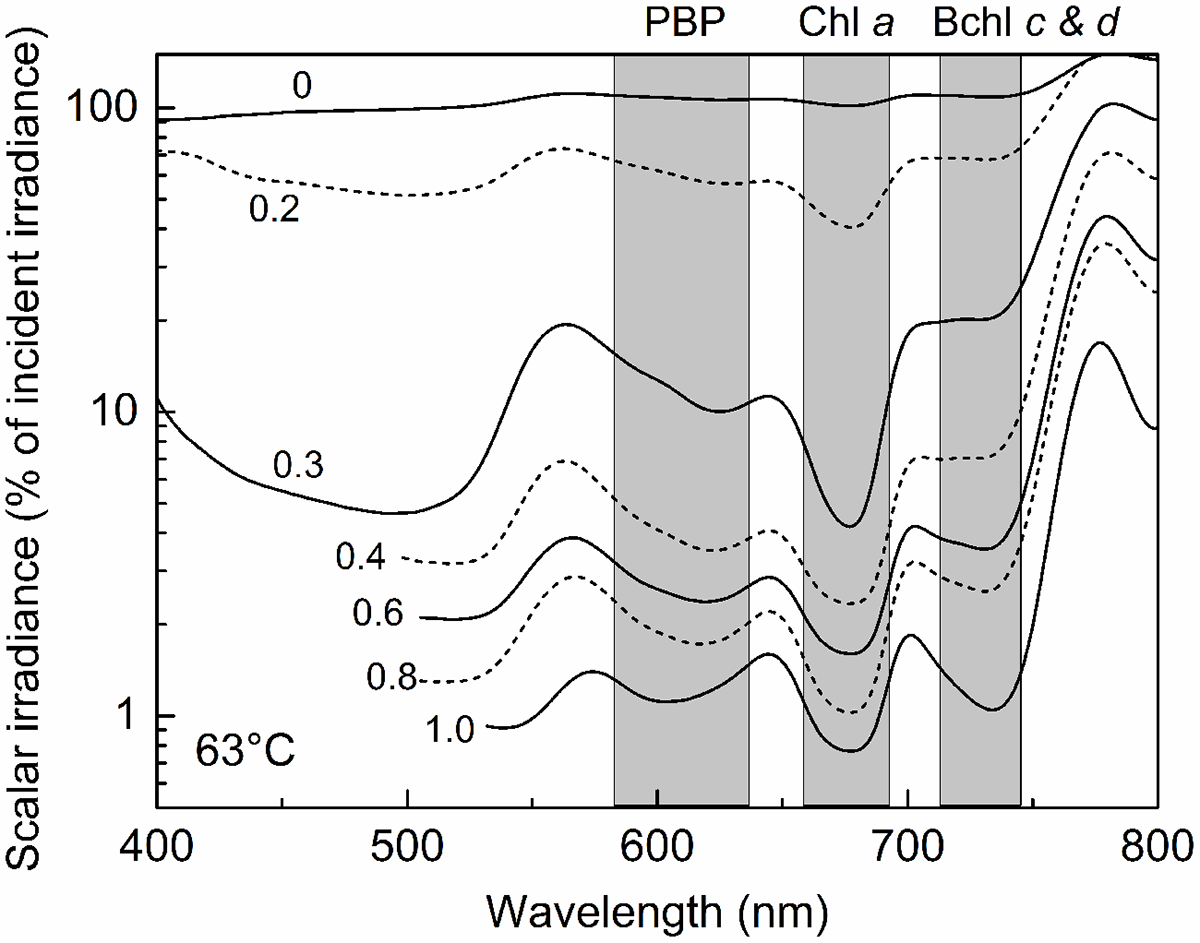
**Spectral scalar irradiance in the 63°C Mushroom Spring mat from September 2008.** Numbers on curves denote the depth (mm) below the mat surface. Note log scale showing transmitted light in % of the downwelling irradiance measured at the mat surface. Shaded areas indicate absorption range of phycocyanin (PC), Chlorophyll (Chl) *a*, and bacteriochlorophylls (Bchl) *c* & *d*.

This stratification paralleled changes in the light environment over the upper 1 mm-thick green surface layer of the mat. Microsensor analyses of scalar irradiance demonstrated that both the intensity and spectral composition changed dramatically with depth in the upper green layer of the mat (**Figure 4**). The uppermost 0.2 mm layer showed moderate attenuation of visible and far-red light, while strong attenuation was found in mat layers 0.2–0.3 mm below the mat surface. The spectral minima indicated presence of chlorophyll *a* (Chl *a*), bacteriochlorophyll *c/d* (Bchl *c*/*d*) and phycobiliproteins (PBPs) as the major pigments. With increasing depth, absorption maxima in the spectral range 600–630 nm, presumably representing phycobiliproteins, showed a distinct blue-shift.

### Canonical Correspondence Analyses

**FIGURE 5:**
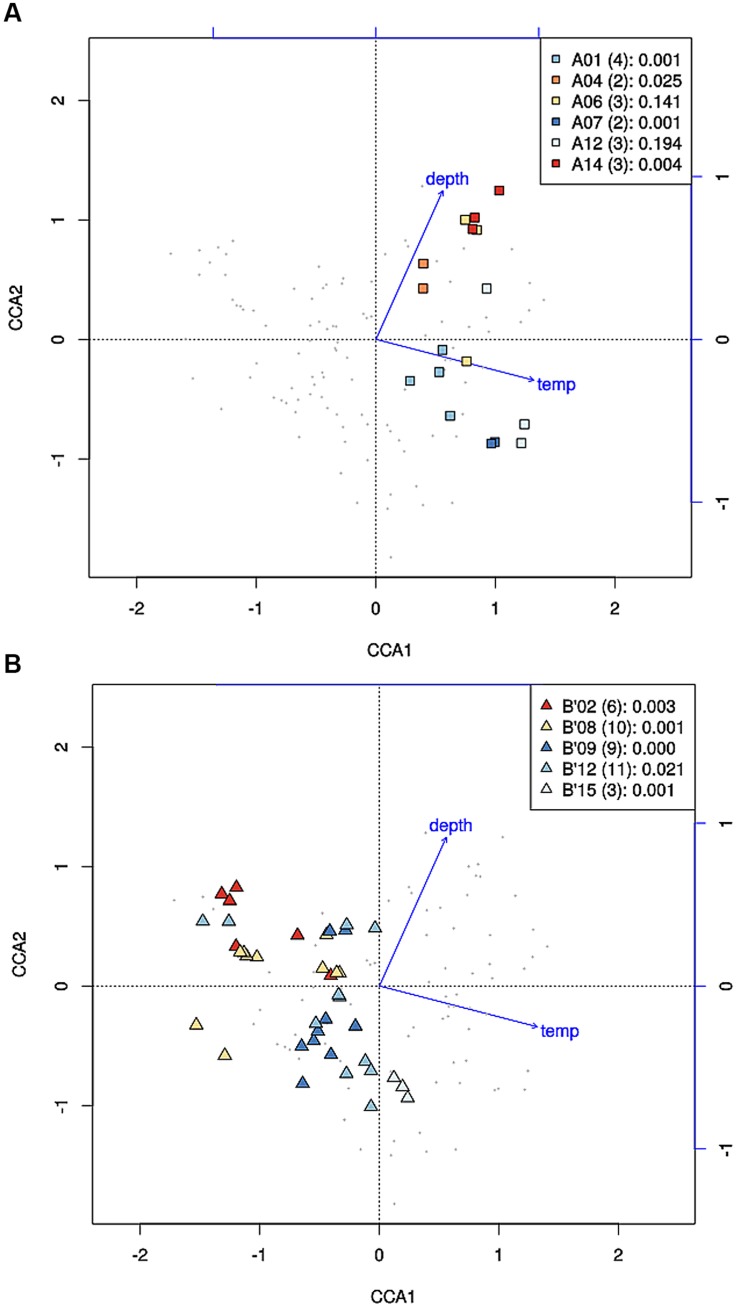
**Canonical correspondence analyses of high-frequency *psaA* sequence variants predicted by Ecotype Simulation to belong to the same targeted putative ecotype (PE) for A-like **(A)** and B′-like **(B)***Synechococcus* PEs.** Small gray data points represent high-frequency sequence variants detected in analysis of six vertical profiles in replicate samples from 60, 63, and 65°C. High-frequency sequences that represent predominant PEs are color-highlighted as indicated by the inset together with the number of variants and the *p*-value associated with the cluster being different from random.

Where high-frequency sequences predicted to belong to the same PE were sufficiently abundant to analyze, they appeared to co-vary along temperature and depth transects (**Figures 2** and **3**). That is, each constituent sequence within a PE changed proportionately with the entire set of sequences from the PE. Environmental associations among the high-frequency sequences within A-like and B′-like PEs recovered from vertical sections of cores collected at 60, 63, and 65°C were tested in a single CCA analysis, and the results for predominant PEs are displayed separately for each lineage in **Figures 5A,B** (see Supplementary Figure S4 for non-predominant PEs, including A′ PEs). The analyses demonstrated strong evidence of clumped distributions of the various high-frequency sequences within each predominant PE, with respect to temperature and depth (Ordtest analyses, *P* < 0.001).

**FIGURE 6:**
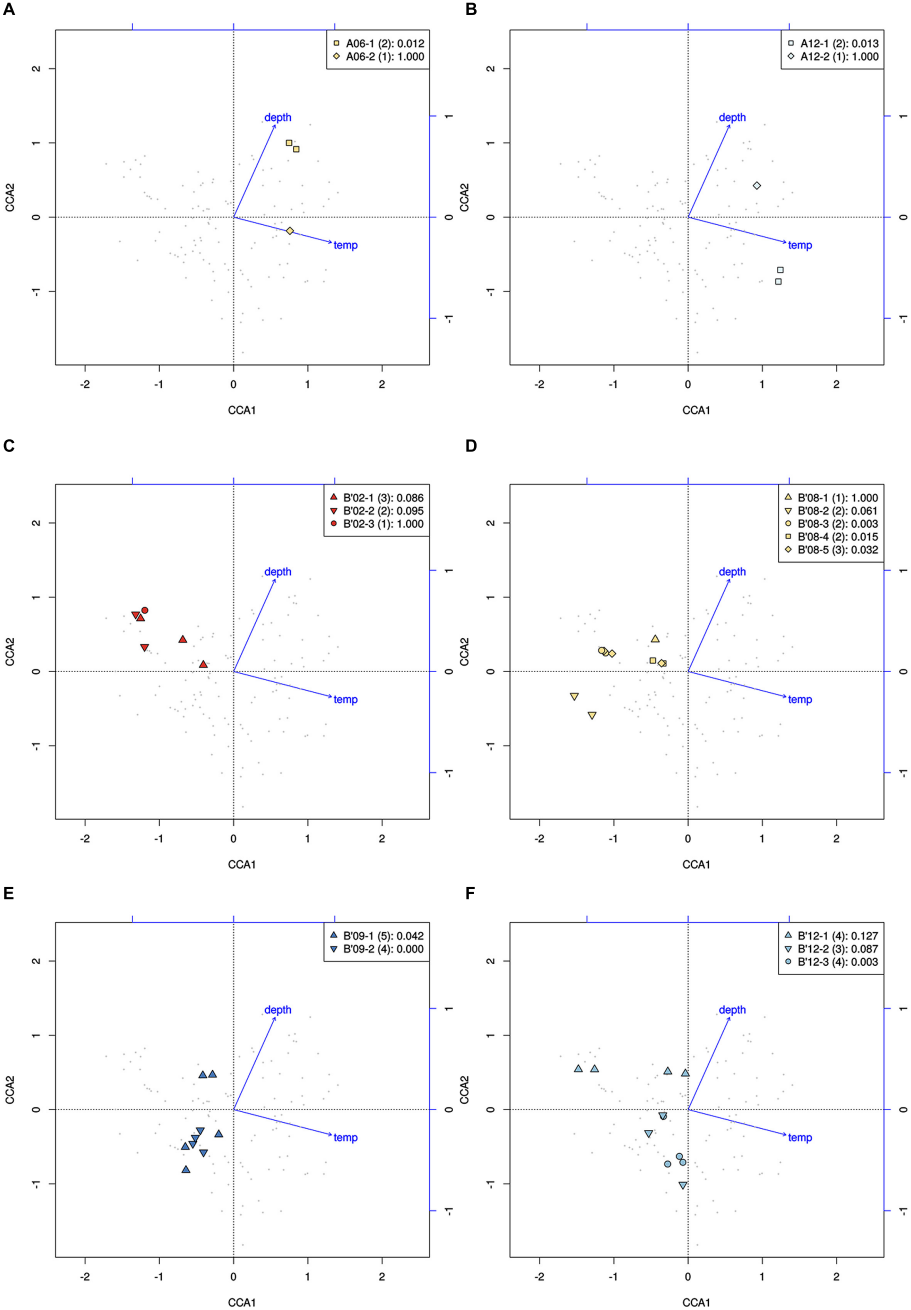
**Canonical correspondence analyses of high-frequency *psaA* sequence variants predicted by Ecotype Simulation to belong to the same PE. (A,B)** Clustering of predominant variants (squares) to the exclusion of the least abundant variant (diamonds) within A-like PEs demarcated using the fine-scale approach. **(C–F)** Clustering of variants within PEs demarcated by the fine-scale approach (different symbols) that were demarcated as a single PE by the conservative approach (all points). Legends indicate how symbols correlate with PEs shown in **Figure 1** and include the *p*-value associated with the cluster being different from random.

High-frequency sequences assigned to most A-like PEs formed clusters based on their temperature and depth distributions that were unlikely to have formed by chance (significance levels for PEs were all *p* ≤ 0.025). This suggested ecological homogeneity among variants within PEs A1, A4, A7, and A14. Variants predicted to comprise PEs A6 and A12 were not significantly clustered (*p* = 0.141 and 0.194), but these PEs each contained three high-frequency sequences, two of which formed a significant cluster to the exclusion of the third variant, which was much more rare (**Figures 6A,B** and see **Figure 1A**). The most abundant PE A6 variants were distributed at depths that overlapped with variants of PE 14. The most abundant PE A12 variants were distributed near the surface of the mat at higher temperatures. All other A-like PEs exhibited unique distributions. PEA7 was found at higher temperatures than PEs A1, A4, A6, and A14, which were distributed with depth in the same order as shown in analyses of all variants within PEs (**Figure 3**). Conservative demarcation lumped clades that were, in some cases (e.g., PEs A6 and A12), ecologically distinct (**Figure 1A**).

Each predominant B′-like PE demarcated using the conservative approach formed a cluster unlikely to have formed by chance (*p* ≤ 0.023; **Figure 5B**), again suggesting ecological homogeneity among variants within all PEs. For example, PE B′15 variants formed a tight cluster distributed toward warmer surface samples. PE B′2 formed a looser cluster distributed in cooler and deeper samples. Fine-scale demarcations permitted us to explore the structure of conservatively demarcated B′-like PEs in greater detail. For instance, fine-scale demarcation predicted that PE B′2 contained three ecotypes (these are designated PE B′2-1, -2, and -3; **Figure 1B**), but CCA analyses did not provide statistical support for this (**Figure 6C**). PE B′9 clustered mainly near the surface of warmer samples, with the exception that two variants were found in deeper samples. Fine-scale demarcation suggested that conservatively demarcated PE B′9 contains two ecotypes (PE B′9- and PE B′9 -2; **Figures 1B and 6E**). PE B′9-2 represented 90% of the population and was surface-associated. PE B′9-1 represented ~10% of the population and contained the two variants that were more deeply distributed and other surface-associated variants, possibly explaining the subsurface bulge in the PE B′9 profile shown in **Figure 3A**.

Putative ecotypes B′8 and B′12 exhibited broader distributions relative to temperature and mat depth. Fine-scale demarcation split these PEs into five and three ecotypes, respectively (**Figure 1B**), many of which clustered uniquely (**Figures 6D,F**). The most abundant fine-scale B′8 PE, PE B′8-4, was distributed in warmer deeper samples and was separated from fine-scale PE B′8-3, which was distributed toward cooler, deeper samples, but the PE B′8-5 distribution overlapped with these two PEs. PE B′8-2 variants formed a cluster that was distributed in cooler surface environments. The one significantly distinct fine-scale B′12 PE, PE B′12-3, was distributed in samples collected in surface layers of warmer mats.

Although coverage was deep, the number of unique high-frequency sequence variants detected per PE was relatively low (i.e., 1–4 for A-like and 1–10 for B′-like PEs). Thus, we also performed CCA analyses in which we included all variants that occurred at least 10 times in the dataset (as opposed to 50 times), increasing the number of variants per PE 2- to 10-fold. Clustering of all predominant PEs remained significant with 54, 23, and 56 variants in the case of PEs A1, B′8-4 and B′9-2, respectively. The only change in results was that subsets of PEs A6 and A12 and fine-scale PE B′8-5 were no longer statistically supported, and subclade B′12-1 became statistically significant in CCA analyses.

CCA also found that high-frequency sequence variants within B′-like, A-like and A′-like PEs were progressively distributed from lower to higher temperatures, respectively (compare **Figures 5A,B** and see Supplementary Figure S4). The vertical stratification of PE B′9, above PE A1 above PE A4 above PEs A6 and A14 was also observed (compare **Figures 5A,B**). Temperature and vertical position explained ~19 and ~8% of the variation in the distribution of PEs among samples, respectively, which suggests that other parameters must also be important in defining the niches of these PEs.

### Responses to Environmental Perturbations

Environmental perturbation studies were used to test whether differences in PE distributions reflect adaptations to different temperature and irradiance levels and whether the high-frequency sequences within a given PE respond homogenously. Results are summarized in **Tables 1** and **2**. As described in Supplementary Section III (Supplementary Figure S5), during similar light alteration experiments in 1996 populations, control samples remained stable over the period of time studied (*p* = 0.35; see Supplementary Table S2). The observed changes in relative abundance (up to fivefold in 2 days) are easily accommodated by observed growth rates of 1–3 doublings per day at optimal temperature and irradiance (Nowack et al., 2015, this issue).

#### Temperature Increase

**FIGURE 7:**
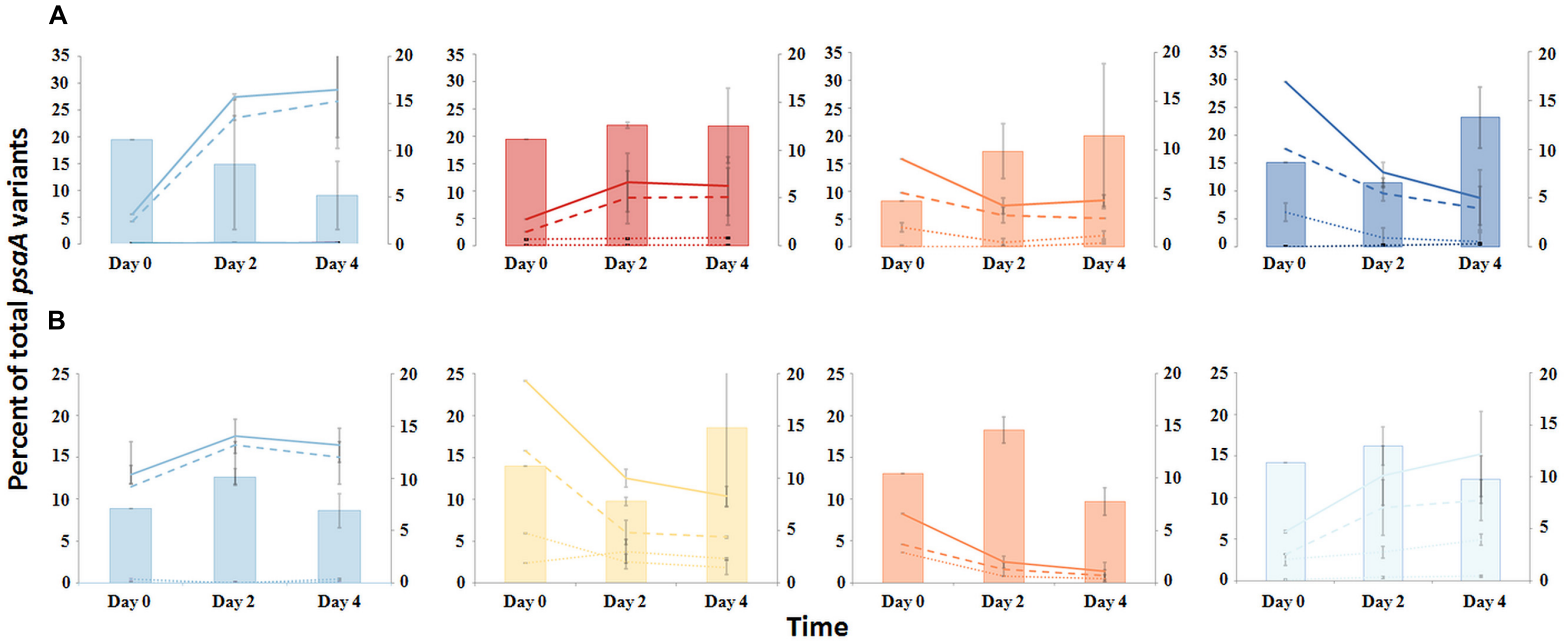
**Responses of PEs to environmental change. (A)** Temperature shift from 60 to 65°C or **(B)** Reduction of solar irradiance by ~92%. Solid lines represent all members of a PE, the dashed line represents the percentage of dominant variant sequences, and dotted lines indicate the percentage of each additional high frequency sequence variant (left axis scale). Percent of low-frequency sequence (LFS) variants in PEs (filled bars; right axis scale). Thin bars indicate the range between replicate samples.

The PE composition changed in the 4 days following a shift of samples collected at a 60°C site to a 65°C site (**Figure 7A**; Supplementary Table S13). The most abundant PEs in the temperature manipulation experiment were significantly different from one another in their responses to increased temperature (ANCOVA test of covariance with time, *P* = 0.0155, *F* = 3.83, *df* = 5.18; see Supplementary Table S2). In particular, PE B′9 declined, while PEs A1 and A14 increased in relative abundance. Effectively, the composition of abundant *Synechococcus* PEs within the sample shifted from that roughly characteristic of the 60°C mat to that roughly characteristic of the 65°C mat, as expected from temperature distributions (Supplementary Table S4; Becraft et al., 2011). While PEs A6 and A14 had similar vertical distributions at 60–63°C sites (**Figure 3**), all variants within PE A6 decreased, while all variants within PE A14 increased after shifting from 60°C to 65°C, suggesting that these PEs have different optimal growth temperatures.

#### Light Alteration

Light alteration experiments were conducted at 63°C in order to focus on very closely related A-like PEs (**Figure 7B**; Supplementary Table S5). The most abundant PEs were significantly different in their responses to removal of 92% of the ambient light (ANCOVA test of covariance with time, *P* = 0.0262; see Supplementary Table S2A). After shading, PEs A4 and A6 declined, whereas PEs A1 and A12 increased in relative abundance (Supplementary Table S14). Thus, while PEs A1 and A4 had similar distributions along the vertical aspect of the mat at 60–63°C sites (**Figure 3**), they had different responses to light reduction.

#### Homogeneity of Responses within PEs

Most high-frequency sequences within a given PE appeared to respond similarly to the perturbations (**Figure 7** and Supplementary Table S2). We tested for homogeneity of response within an abundant PE by addressing whether the pool of all low-frequency sequences changed its relative frequency within the PE after an environmental perturbation. The proportion of low-frequency sequence variants among all sequences within a PE changed little over the environmental perturbation (by 0.3–7.6%) (generalized linear model analysis, *P* > 0.34; see Supplementary Table S3), despite 1.5- to 5-fold increases or decreases in PE relative abundances in response to environmental perturbations (**Figures 7A,B**).

### Transcription Patterns

**FIGURE 8:**
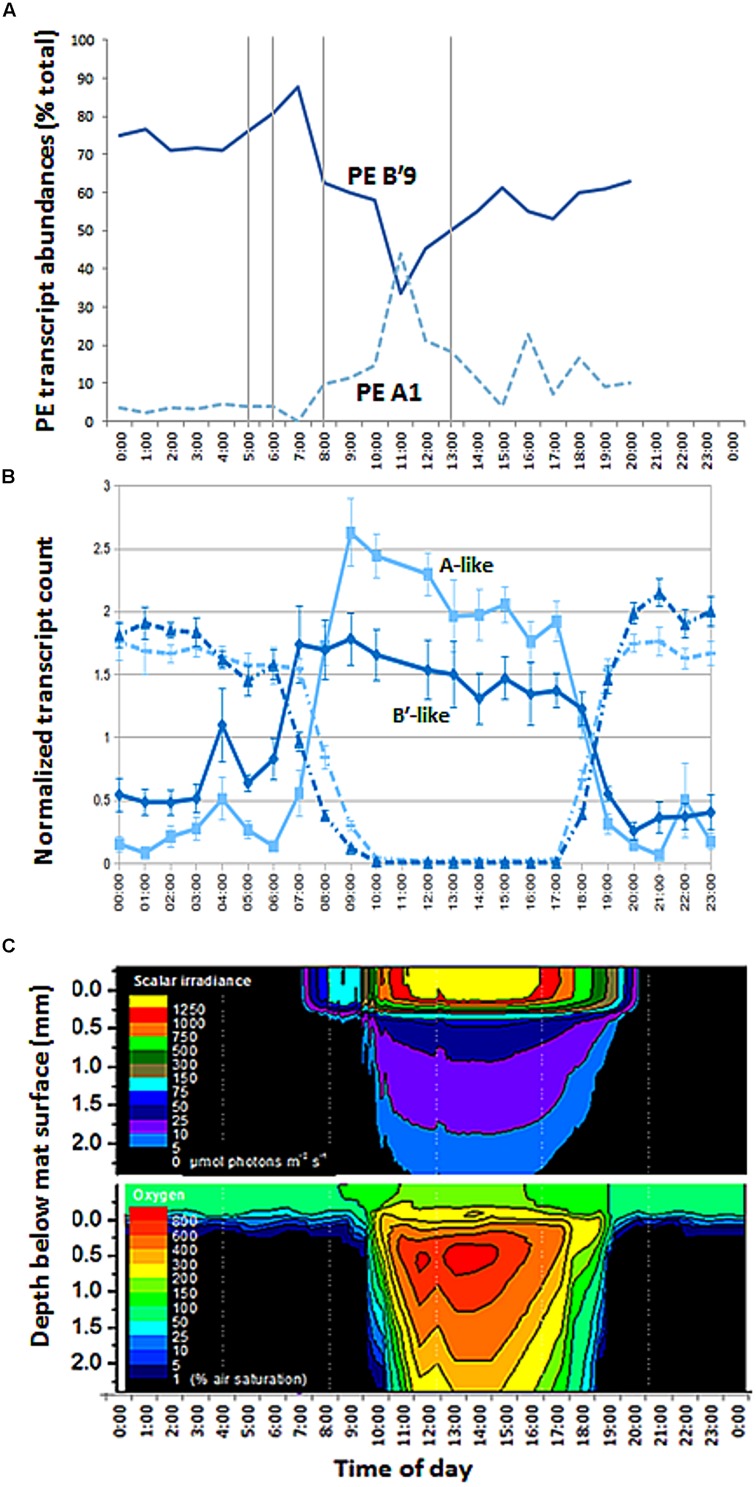
**Diel transcription and change in irradiance and oxygen concentration with depth in 60°C Mushroom Spring mat. (A)** Relative abundances of transcripts of abundant PEs B′9 and A1 measured from *psaA* cDNA over the diel cycle. Samples corresponding to time points at 0500, 0600, 0800, 1300 (vertical lines), 2100, 2200, and 2300 are missing due to failed sequencing reactions. **(B)** Normalized count of B′-like (dark blue) and A-like (light blue) combined photosystem (solid line) and nitrogen fixation (dashed line) transcripts over the diel cycle. **(C)** Isopleth diagrams showing depth distribution of scalar irradiance (μmol photons m^-2^ s^-1^) and O_2_ concentration (% air saturation) over the diel cycle. Samples at time points 1700, 1800, 1900, and 2000 h in part A were from the previous evening, 11 September 2009.

Sequencing of *psaA* transcripts from 60°C samples collected over a diel cycle demonstrated differential expression of transcripts contributed by the dominant *Synechococcus* PEs B′9 and A1 (**Figure 8A**; *G*-test, *P* < 0.001; Supplementary Figure S3 and Table S1). The expression of results as relative percentages limited our understanding of the basis for these differences. However, we were able to use the genome sequences of isolates representative of PEs *Synechococcus* B′ and A to recruit separately A-like and B′-like transcripts of photosynthesis and N_2_ fixation genes from a metatranscriptome of this mat produced over the same diel cycle (Liu et al., 2012). In the case of photosynthesis genes, B′-like transcripts were expressed before A-like transcripts (**Figure 8B**). Because both B′-like and A-like photosynthesis genes were transcribed diurnally, and because PE B′9 and PE A1 transcripts dominated the pools of B′-like and A-like *psaA* transcripts, the decreased relative abundance of PE B′9 transcripts in mid-day (**Figure 8A**) was apparently due to the increase in relative abundance of PE A1 transcripts. Dominance of PE B′9 transcripts at night was observed in the data on relative abundance, but *psaA* transcripts in general were low at night (**Figure 8B**). A similar offset was observed in B′-like and A-like nitrogen fixation gene transcription.

The later transcription of PE A1 photosynthesis genes may result from the limited penetration of light into the mat until between 1000 and 1100 h, when irradiance reached 5–100 μmol photons m^-2^ s^-1^ between 320 and 720 μm below the mat surface (**Figure 8C**), the depth where this population was predominantly located (**Figure 3**). Oxygen concentrations at these mat depths increased between 1000 and 1100 h reaching 3- to 6-times air saturation, and further accumulated to >8 times air saturation by 1300 h, which reflects increased photosynthetic activity at greater depth. In contrast, photon irradiance increased to between ~500 and 1250 μmol photons m^-2^ s^-1^ at the mat surface between 1000 and 1100 h in parallel with a decline in PE B′9 transcript relative abundance.

## Discussion

Next-generation sequencing technology allowed deep coverage of a broad array of environmental samples, and this has allowed us to demonstrate the ecological distinctions of most of the predominant sequence clusters predicted to be PEs by Ecotype Simulation. Importantly, the deep coverage provided access to sequence variation within PEs, allowing us to test whether the variants predicted by Ecotype Simulation to comprise a given PE are equivalently distributed along ecological gradients and behave equivalently in response to environmental change (i.e., they are ecologically interchangeable).

Pyrosequencing showed that PEs collected along the thermal gradient in the Mushroom Spring effluent channel in 2008 differed in their temperature ranges, as suggested by our previous studies of samples collected in 2006 and analyzed by cloning and sequencing (**Tables 1** and **2**; Supplementary Table S4). However, sequencing and CCA analyses in the present study further demonstrated that the variants predicted to belong to the same PE were distributed similarly, indicating ecological homogeneity within most PEs. PEs also responded differently to shifts in temperature, providing further evidence of divergence in their temperature preferences, and these shifts were consistent with temperature adaptations of cultivated *Synechococcus* strains (Miller and Castenholz, 2000; Allewalt et al., 2006). Furthermore, these experiments provided additional evidence that all of the members predicted to belong to the same PE respond to temperature in concert.

Pyrosequencing analyses also allowed us to resolve PEs that were distributed uniquely in the vertical aspect of the 60–63°C mat. CCA analyses demonstrated that all variants predicted to belong to the same PE were distributed similarly. These findings significantly extend earlier observations, in which vertical positioning of PEs was obscured either by the use of slowly evolving molecular markers, such as 16S rRNA and the 16S–23S rRNA internal transcribed spacer region (Ramsing et al., 2000; Ferris et al., 2003) or by the inability of DGGE to discern closely related sequence variants (Becraft et al., 2011). The light environment changes significantly over the top 1 mm of the microbial mat both with depth and over time (**Figure 8C**). The uppermost 0.1–0.2 mm experience high photon irradiance (>200–2000 μmol photons m^-2^ s^-1^) for most of the sun-exposed part of the day, while deeper mat layers (>0.5 mm depths) experience much lower light levels (maximally ~50 μmol photons m^-2^ s^-1^ during mid-day), and an altered light spectrum, due to the strong attenuation and scattering of visible wavelengths, and oxygenic photosynthesis in these mat layers thus remains light limited throughout most of the day (**Figure 8C**). PEs responded differently to reduced irradiance, suggesting that adaptation to light may, at least in part, underlie these distribution patterns, and similar responses of all members of each PE again suggested ecological homogeneity of PE populations. Indeed, in other studies reported in the second paper of this series (Nowack et al., 2015, this issue), we have observed that *Synechococcus* strains representative of some of the PEs residing in deeper parts of the mat green layer have adaptations to low irradiance that confer the ability to acclimate to low irradiance values that are consistent with the light levels present at these depths.

Our observation that PE B′9 transcripts were expressed earlier than PE A1 transcripts matches the relatively longer and shorter light exposure periods experienced by these surface- and subsurface-associated populations, respectively (**Figure 8C**). Compared to deeper, subsurface populations (e.g. PE A1), the surface localization of PE B′9 might suggest that genes encoding its photosynthetic apparatus need to be transcribed earlier (i.e., before sunrise) to be available to harvest light energy in the morning. Interestingly, previous *in situ* microsensor studies showed that peak oxygenic photosynthesis moved downward to deeper portions of the 1 mm-thick upper green *Synechococcus* layer as light intensity increased during the morning (Ramsing et al., 2000). This might reflect the increased activity of deeper *Synechococcus* PEs, possibly combined with inhibition of photosynthesis in PEs nearer the mat surface when irradiance is highest.

While pyrosequencing provided an opportunity to examine the ecological interchangeability of individual variants within PEs, these results must be interpreted with caution. At issue is whether the resolution provided by a single gene, in this case *psaA*, can identify the most newly divergent ecotypes; also at issue is the appropriate coarseness of demarcation by Ecotype Simulation. A conservative demarcation of a PE might yield an ecologically heterogeneous set of variants, which could comprise multiple ecotypes instead of just one. It appears that conservative demarcation of several B′-like PEs yielded an ecologically heterogeneous set of organisms. For example, fine-scale demarcation of PE B′8 yielded PEs B′8-3 and B′8-4, which are significantly different in their temperature and depth distributions. In other cases, such as PE B′8-5, PEs cannot be resolved from others by their temperature and depth distributions, and so unmeasured parameters may explain their distinctions and their ability to co-exist. Comparative genomic analyses reported in the third paper of this series (see Olsen et al., 2015, this issue) are beginning to provide insights into such unsuspected adaptations.

In principle, complete ecological interchangeability is expected only in the populations that have descended from an ancestor that survived the most recent periodic selection event and have not subsequently split to form a new ecologically distinct population. The need to find the smallest clades with ecologically interchangeable membership emphasizes the importance of molecular resolution. For instance, even fine-scale PE demarcation yielded extremely closely related populations within PEs A6 and A12, which might prove to be the ecologically homogeneous ecotypes.

The deeper coverage of genetic variants and habitats provided by pyrosequencing analyses resulted in the demarcation by Ecotype Simulation of a greater number of non-singleton PEs than before. Whereas Becraft et al. (2011) used the conservative approach to detect 7 A-like and 12 B′-like PEs, here we detected 11 A-like 18 B′-like PEs. Although ~500 times more sequences were included in the present study, all the high-frequency sequences found in the present study were detected previously in our smaller samples. This implies that the greater number of PEs demarcated resulted from the deeper coverage provided by pyrosequencing rather than changes in PE abundances in the mat between 2006 and 2008. Some PEs that were newly predicted in the present analyses corresponded to subclades previously embedded within PEs that had not been identified as PEs from more limited clone sequence data (e.g., PE A12; as shown in **Tables 1** and **2**), or to low-abundance populations not detected in clone libraries (e.g., PE A10). Many of these newly discovered PEs appear to be comprised of ecologically homogeneous members (see Supplementary Figure S4). Although cloning and sequencing studies analyzed only 0.2% of the amount of sequence data obtained in pyrosequencing study of samples collected in 2008, this approach still detected 66% of the currently demarcated PEs. In addition, newly demarcated PEs generally exhibited low relative abundance; this implies that the predominant *Synechococcus* populations contributing to the environment in these habitats have confidently been identified.

In summary, our analysis of sequence variation of an essential protein-encoding locus identified populations that meet the expectations of ecological species: (i) they are ecologically distinct populations and (ii) as shown by the similarity of their environmental distributions and their responses to environmental perturbations, the memberships of many predicted ecological species are ecologically interchangeable. These and other results (Melendrez et al., 2011) indicate that molecular divergence among ecological species is much less than among the species recognized by microbial systematists, including definitions of 1–3% 16S rRNA divergence (Wayne et al., 1987; Stackebrandt and Ebers, 2006) or >95% (Konstantinidis and Tiedje, 2005; Konstantinidis, 2011) or 95–96% (Chun and Rainey, 2014; Kim et al., 2014) average nucleotide identity (ANI). As will be demonstrated in the accompanying papers (Nowack et al., 2015, this issue; Olsen et al., 2015, this issue), *psaA* was able to detect species that were identical or nearly identical (0.06% divergent) in their full-length 16S rRNA gene sequences and >98.35% identical in their average nucleotide indeces. In these and other habitats, where microbial diversity is organized by physico-chemical parameters, ecological species populations are likely to be the fundamental units of community composition and structure. If the level of molecular resolution needed to detect species in the communities we have studied is typical, the widespread use of high-throughput 16S rRNA sequencing methods may be masking the true species diversity and dynamics across microbial communities in a wide variety of habitats.

## Conflict of Interest Statement

The authors declare that the research was conducted in the absence of any commercial or financial relationships that could be construed as a potential conflict of interest.

## Footnotes

1 http://454.com/downloads/my454/documentation/gs-flx-plus/Rapid-Library-Preparation-Method-Manual_XLPlus_May2011.pdf.

2 http://github.com/sandain/pigeon

3 http://fcohan.web.wesleyan.edu/ecosim/
