# Supplementary material for: The molecular dimension of microbial species: 1. Ecological distinctions among, and homogeneity within, putative ecotypes of Synechococcus inhabiting the cyanobacterial mat of Mushroom Spring, Yellowstone National Park
Source: Front Microbiol. 2015 Jun 22;6:590. doi: 10.3389/fmicb.2015.00590 (PMC4475828; doi:10.3389/fmicb.2015.00590)
Supplement: Supplementary file 1 [file Data_Sheet_1.DOC]

8. Supplementary information for the article entitled:

**Ecological distinctions among, and homogeneity within, putative ecotypes of *Synechococcus* inhabiting the cyanobacterial mat of Mushroom Spring, Yellowstone National Park**

**Eric D. Becraft, Jason M. Wood, Doug B. Rusch, Michael Kühl, Sheila I. Jensen, Donald A. Bryant, David W. Roberts, Frederick M. Cohan and David M. Ward**

TABLE OF CONTENTS

Section I. Positioning of the *psaA* region studied relative to proteins in the photosystem I complex. 3

Section II. Code for CCA-plot.R script 4

Section III. Controls for light reduction experiment 10

Supplementary References 10

Supplementary Tables**:**

**Table 1:** G-test P–values and statistics for all distribution experiments. 11

**Table 2:** anova P–values and statistics for light reduction temperature shift experiments. 12

**Table 3:** P–values for within-PE population genetics measured over the four day light reduction experiment and temperature shift experiment. 13

**Table 4:** PE population percentages along replicate effluent flow path samples. 14

**Table 5:** Percent population of A-like, A′-like and B′-like PEs in pooled vertical cores in relation to whole temperature samples. 15

**Table 6:** PE population percentages along the vertical gradient at ~60°C site. 16

**Table 7:** PE population percentages along the vertical gradient at ~60°C site. 17

**Table 8:** PE population percentages along the vertical gradient at ~63°C site. 18

**Table 9:** PE population percentages along the vertical gradient at ~63°C site. 19

**Table 10:** PE population percentages along the vertical gradient at ~65°C site. 20

**Table 11:** PE population percentages along the vertical gradient at ~65°C site. 21

**Table 12:** PE population percentages changing temporally during the temperature shift from 60°C to 63°C. 22

**Table 13:** PE population percentages over time during the light alteration experiments conducted in 63°C. 23

**Supplementary Figures**:

**Figure 1: Photographs of the temperature shift experiment and light alteration covers.** 24

**Figure 2:** Subunits of the Photosystem I complex of *Synechococcus elongatus*. 25

**Figure 3:** Frequency plots of *psaA* variants predicted to belong to the same putative ecotype populations**.** 26

**Figure 4:** Canonical correspondence analyses of high-frequency *psaA* sequence variants predicted by Ecotype Simulation to belong to abundant putative ecotypes. 28

**Figure 5:** Change in relative abundance of abundant PEs in undisturbed experiment in 1996 in Mushroom Spring mat at 60°C.. 30

**Section I. Positioning of the *psaA* region studied relative to proteins in the photosystem I complex**

Becraft et al. (2011) used primers *psaA*forward 5'-CTGAGCGGCATGTACTACCA-3' and *psaA*reverse 5'- CAGGCCACCCTTGAAGGTG-3' to produce 523-bp amplicons of *psaA* for DGGE analysis, cloning and sequencing. This corresponds to amino acid residues 87 to 285 of PsaA. In the current study, the forward primer was changed to *psaA*centerforward 5'-TTCCACTACCACAAGCGGGCTCC-3' and used with the same *psaA*reverse primer to produce a smaller, overlapping amplicon of 325 bp, which corresponds to amino acid residues 178 to 285 of PsaA. This region corresponds to the last three amino acid residues of transmembrane helix 2, a small stromal loop, transmembrane helix 3, and most of the lumenal loop that connects helices 3 and 4 (see Supplemental Figure 2). Much of this region of the protein is surface-exposed, does not interact with any electron transfer partner protein, and has only a very limited contact region with the non-essential subunit, PsaK (see Supplemental Figure 2). This region binds two β-carotene molecules and contains seven conserved histidine residues, all seven of which bind chlorophyll *a* molecules. However, overall this region is probably as structurally unconstrained as any region of Photosystem I, and thus the region of *psaA* should not be under strong selection pressure other than for retention of the seven chlorophyll-ligating histidine residues. This is especially true for the lumenally exposed loop (residues 221 to 285), where many nucleotide changes that partly define the putative ecotypes occur.

**Section II: Code for CCA-plot.R script**

#!/usr/bin/Rscript

suppressPackageStartupMessages **(**

library **(**vegan**)**

**)**

usage **<-** "Usage: <Community Matrix> <Constraining Matrix> <Conditioning Matrix> <Community Categories> <Sample Categories> <Output File> <Legend Location>"

# Custom version of Dave Robert's ordtest {labdsv} code, used to test all data points.

ordtest **<-** **function** **(**ordpoints, var, dim **=** c **(**1**:**ncol **(**ordpoints**))**, index **=** 'euclidean', nitr **=** 1000**)** **{**

tdist **<-** 0

observed **<-** 0

reps **<-** rep **(**0, nitr **-** 1**)**

var **<-** factor **(**var**)**

**for** **(**i **in** levels **(**var**))** **{**

mask **<-** var **==** i

tdist **<-** tdist **+** sum **(**dist **(**ordpoints**[**mask,dim**]**, index**))**

**}**

observed **<-** tdist

**for** **(**i **in** 1**:(**nitr **-** 1**))** **{**

tdist **<-** 0

var **<-** sample **(**var, length **(**var**)**, replace **=** **FALSE)**

**for** **(**j **in** levels **(**var**))** **{**

mask **<-** var **==** j

tdist **<-** tdist **+** sum **(**dist **(**ordpoints**[**mask, dim**]**, index**))**

**}**

reps**[**i**]** **<-** tdist

**}**

reps

out **<-** list **(**obs **=** observed, p **=** **(**sum **(**reps **<=** observed**)** **+** 1**)** **/** nitr, reps **=** reps**)**

out

**}**

# Dave Robert's alt.ordtest, used to test each cluster of points.

alt.ordtest **<-** **function** **(**ord, var, index **=** "euclidean", nitr **=** 1000**)** **{**

**if** **(**sum **(**var**)** **<=** 1**)** **{**

out **<-** list **(**obs **=** 0, p **=** 1, reps **=** rep **(**1, nitr **-** 1**))**

**}**

**else** **{**

tdist **<-** 0

observed **<-** 0

reps **<-** rep **(**0, nitr **-** 1**)**

tdist **<-** sum **(**dist **(**ord**$**species**[**var,**]**, index**))**

observed **<-** tdist

**for** **(**i **in** 1**:(**nitr **-** 1**))** **{**

tdist **<-** 0

var **<-** sample **(**var, length **(**var**)**, replace **=** **FALSE)**

tdist **<-** sum **(**dist **(**ord**$**species**[**var,**]**, index**))**

reps**[**i**]** **<-** tdist

**}**

out **<-** list **(**obs **=** observed, p **=** **(**sum **(**reps **<=** observed**)** **+** 1**)** **/** nitr, reps **=** reps**)**

**}**

out

**}**

# This was a private method used by the plot.cca {vegan} function that was

# copy-pasted for use here.

#

# Scaling of arrows to 'fill' a plot with vectors centred at 'at'.

# Plot dims from 'par(\"usr\")' and arrow heads are in 'x'.

ordiArrowMul **<-** **function** **(**x, at **=** c **(**0,0**)**, fill **=** 0.75**)** **{**

u **<-** par **(**'usr'**)**

u **<-** u **-** rep **(**at, each**=**2**)**

r **<-** c **(**range **(**x**[**, 1**]**, na.rm **=** **TRUE)**, range **(**x**[**, 2**]**, na.rm **=** **TRUE))**

## 'rev' takes care of reversed axes like xlim(1,-1)

rev **<-** sign **(**diff **(**u**))[-**2**]**

**if** **(**rev**[**1**]** **<** 0**)**

u**[**1**:**2**]** **<-** u**[**2**:**1**]**

**if** **(**rev**[**2**]** **<** 0**)**

u**[**3**:**4**]** **<-** u**[**4**:**3**]**

u **<-** u **/** r

u **<-** u**[**is.finite **(**u**)** **&** u **>** 0**]**

fill ***** min **(**u**)**

**}**

# This was a private method used by the plot.cca {vegan} function that was

# copy-pasted for use here.

#

# Location of the text at the point of the arrow. 'vect' are the

# coordinates of the arrow heads, and 'labels' are the text used to

# label these heads, '...' passes arguments (such as 'cex') to

# strwidth() and strheight().

ordiArrowTextXY **<-** **function** **(**vect, labels, ...**)** **{**

w **<-** strwidth **(**labels, ...**)**

h **<-** strheight **(**labels, ...**)**

## slope of arrows

b **<-** vect**[**, 2**]** **/** vect**[**, 1**]**

## offset based on string dimensions

off **<-** cbind **(**sign **(**vect**[**, 1**])** ***** **(**w **/** 2 **+** h **/** 4**)**, 0.75 ***** h ***** sign **(**vect**[**, 2**]))**

## move the centre of the string to the continuation of the arrow

**for(**i **in** 1**:**nrow **(**vect**))** **{**

move **<-** off**[**i, 2**]** **/** b**[**i**]**

## arrow points to the top/bottom of the text box

**if** **(**is.finite **(**move**)** **&&** abs **(**move**)** **<=** abs **(**off**[**i, 1**]))**

off**[**i, 1**]** **<-** move

**else** **{**

## arrow points to a side of the text box

move **<-** b**[**i**]** ***** off**[**i, 1**]**

off**[**i, 2**]** **<-** move

**}**

**}**

off **+** vect

**}**

# Draw arrows on the ordiplot. Based on code found in the plot.cca {vegan} function.

drawArrows **<-** **function** **(**ord, col **=** 'blue', cex **=** 1.0**)** **{**

**if** **(!** inherits **(**ord, c **(**"ordiplot"**)))**

stop **(**"drawArrows is only defined for ordiplot objects"**)**

draw.mul **<-** ordiArrowMul **(**ord**$**biplot, fill **=** 0.5**)**

attr **(**ord**$**biplot, 'arrow.mul'**)** **<-** draw.mul

arrows **(**0, 0, draw.mul ***** ord**$**biplot**[**, 1**]**, draw.mul ***** ord**$**biplot**[**, 2**]**, length **=** 0.05, col **=** col**)**

axis **(**3, at **=** c **(-**draw.mul, 0, draw.mul**)**, labels **=** rep **(**'', 3**)**, col **=** col**)**

axis **(**4, at **=** c **(-**draw.mul, 0, draw.mul**)**, labels **=** c **(-**1, 0, 1**)**, col **=** col**)**

draw.biplabs **<-** ordiArrowTextXY **(**draw.mul ***** ord**$**biplot, rownames **(**ord**$**biplot**)**, cex **=** cex**)**

text **(**draw.biplabs, rownames **(**ord**$**biplot**)**, col **=** col, cex **=** cex**)**

**}**

# Draw the legend on the ordiplot.

drawLegend **<-** **function** **(**x, y, ord, categories, cex **=** 1.0, ncol **=** 1**)** **{**

**if** **(!** inherits **(**ord, c **(**"ordiplot"**)))**

stop **(**"drawLegend is only defined for ordiplot objects"**)**

# Grab a list of the unique community categories for the legend.

draw.legend **<-** categories**[!** duplicated **(**categories**$**category**)**,**]**

# The cex column and rownames are not used for the legend.

draw.legend**$**cex **<-** **NULL**

rownames **(**draw.legend**)** **<-** **NULL**

# Build the legend.

draw.legend**$**text **<-** rep **(**"", times **=** nrow **(**draw.legend**))**

**for** **(**i **in** 1**:**nrow **(**draw.legend**))** **{**

# Calculate the number of HFS that belong to category i.

n **<-** sum **(**categories**$**category **==** draw.legend**$**category**[**i**])**

# Calculate the p-value using Dave Robert's alt.ortest for category i.

dev **<-** categories**[**rownames **(**ord**$**species**)**, 'category'**]** **==** draw.legend**$**category**[**i**]**

dev**[**is.na **(**dev**)]** **<-** **FALSE**

aot **<-** alt.ordtest **(**ord, dev, nitr **=** 10000**)**

# Create the legend for category i.

draw.legend**$**text**[**i**]** **<-** sprintf **(**"%s (%d): %.3f", draw.legend**$**category**[**i**]**, n, aot**$**p**)**

**}**

# Sort the legend.

draw.legend **<-** draw.legend**[**with **(**draw.legend, order **(**category**))**,**]**

# Draw the legend.

legend **(**

x, y,

legend **=** draw.legend**$**text,

pch **=** as.vector **(**draw.legend**$**pch**)**,

col **=** as.vector **(**draw.legend**$**col**)**,

pt.bg **=** as.vector **(**draw.legend**$**bg**)**,

cex **=** cex,

ncol **=** ncol

**)**

**}**

# Draw the species plot using the plot.cca {vegan} function as a base.

drawSpeciesPlot **<-** **function** **(**

x,

categories **=** data.frame **(**

row.names **=** rownames **(**x**$**CCA**$**v**)**, category **=** rep **(NA**, nrow **(**x**$**CCA**$**v**))**,

pch **=** rep **(**1, nrow **(**x**$**CCA**$**v**))**, col **=** rep **(**'black', nrow **(**x**$**CCA**$**v**))**,

bg **=** rep **(NA**, nrow **(**x**$**CCA**$**v**))**, cex **=** rep **(**1.0, nrow **(**x**$**CCA**$**v**))**

**)**,

lg.x **=** 'bottomleft',

lg.y **=** 'NULL',

scaling **=** 2,

cex **=** 1.0

**)** **{**

**if** **(!** inherits **(**x, c **(**"cca"**)))**

stop **(**"drawPlot is only defined for cca objects"**)**

# Draw the plot.

p **<-** draw.plot **<-** plot **(**x, type **=** 'none', scaling **=** scaling**)**

# Draw the axis and arrows for the constraining variables.

drawArrows **(**draw.plot, col **=** 'blue', cex **=** cex**)**

# Draw small gray dots for all of the species points.

points **(**draw.plot**$**species, pch **=** 3, col **=** 'gray', cex **=** 0.2**)**

# Draw all points defined in the categories matrix.

**for** **(**i **in** 1**:**nrow **(**categories**))** **{**

draw.cat **<-** categories**[**i,**]**

points **(**

draw.plot**$**species**[**rownames **(**draw.cat**)**, 1**]**,

draw.plot**$**species**[**rownames **(**draw.cat**)**, 2**]**,

pch **=** as.vector **(**draw.cat**$**pch**)**,

col **=** as.vector **(**draw.cat**$**col**)**,

bg **=** as.vector **(**draw.cat**$**bg**)**,

cex **=** as.vector **(**draw.cat**$**cex**)**

**)**

**}**

# Draw the legend.

**if** **(!** all **(**is.na **(**categories**$**category**)))** **{**

drawLegend **(**lg.x, lg.y, draw.plot, categories, cex **=** cex**)**

**}**

p

**}**

# Load the command line arguments.

args **<-** commandArgs **(**trailingOnly **=** **TRUE)**

# Verify the command line arguments.

**if** **(**length **(**args**)** **<** 6**)** stop **(**usage**)**

communityMatrixFile **<-** args**[**1**]**

constrainingMatrixFile **<-** args**[**2**]**

conditioningMatrixFile **<-** args**[**3**]**

sampleCategoriesFile **<-** args**[**4**]**

communityCategoriesFile **<-** args**[**5**]**

outputFile **<-** args**[**6**]**

legendLocation **<-** args**[**7**]**

# Verify the input files exist.

**if** **(!** file.exists **(**communityMatrixFile**))** stop **(**"Community Matrix file not found."**)**

**if** **(!** file.exists **(**constrainingMatrixFile**))** stop **(**"Constraining Matrix file not found."**)**

**if** **(!** file.exists **(**conditioningMatrixFile**))** stop **(**"Conditioning Matrix file not found."**)**

**if** **(!** file.exists **(**communityCategoriesFile**))** stop **(**"Community Categories file not found."**)**

**if** **(!** file.exists **(**sampleCategoriesFile**))** stop **(**"Sample Categories file not found."**)**

# Load the data files.

community_matrix **<-** read.table **(**communityMatrixFile, header **=** **TRUE)**

constraining_matrix **<-** read.table **(**constrainingMatrixFile, header **=** **TRUE)**

conditioning_matrix **<-** read.table **(**conditioningMatrixFile, header **=** **TRUE)**

community_categories **<-** read.table **(**communityCategoriesFile, header **=** **TRUE)**

sample_categories **<-** read.table **(**sampleCategoriesFile, header **=** **TRUE)**

## Remove rows not found in the sample categories matrix.

community_matrix **<-** community_matrix**[**rownames **(**sample_categories**)**,, drop **=** **FALSE]**

# Remove empty rows and columns from the community matrix.

community_matrix **<-** community_matrix**[**rowSums **(**community_matrix**)** **>** 0,, drop **=** **FALSE]**

community_matrix **<-** community_matrix**[**, colSums **(**community_matrix**)** **>** 0, drop **=** **FALSE]**

# Remove rows and columns not in the community matrix.

constraining_matrix **<-** constraining_matrix**[**rownames **(**community_matrix**)**,, drop **=** **FALSE]**

conditioning_matrix **<-** conditioning_matrix**[**rownames **(**community_matrix**)**,, drop **=** **FALSE]**

community_categories **<-** community_categories**[**colnames **(**community_matrix**)**,, drop **=** **FALSE]**

sample_categories **<-** sample_categories**[**rownames **(**community_matrix**)**,,drop **=** **FALSE]**

community_categories **<-** community_categories**[!**is.na **(**community_categories**[**, 'category'**])**,**]**

# Make sure the community_matrix is the same size as the constraining matrix.

**if** **(!** identical **(**row.names **(**community_matrix**)**, row.names **(**constraining_matrix**)))** **{**

stop **(**"Community and Constraining matrices don't have identical names."**)**

**}**

# Make sure the community_matrix is the same size as the conditioning matrix.

**if** **(!** identical **(**row.names **(**community_matrix**)**, row.names **(**conditioning_matrix**)))** **{**

stop **(**"Community and Conditioning matrices don't have identical names."**)**

**}**

# Make sure the community_matrix is the same size as the sample matrix.

**if** **(!** identical **(**row.names **(**community_matrix**)**, row.names **(**sample_categories**)))** **{**

stop **(**"Community Matrix and Sample Categories don't have identical names."**)**

**}**

# Run the Canonical Correspondence Analysis.

x **<-** cca **(**community_matrix, constraining_matrix**)**

# Output the cca text.

print **(**x**)**

# Save the species plot to a file.

**if** **(**grepl **(**".png$", outputFile**))** png **(**outputFile**)**

**if** **(**grepl **(**".pdf$", outputFile**))** pdf **(**outputFile**)**

**if** **(**grepl **(**".svg$", outputFile**))** svg **(**outputFile**)**

# Draw the plot.

p **<-** drawSpeciesPlot **(**x, community_categories, legendLocation, scaling **=** 3, cex **=** 1.25**)**

# Run the modified version of Dave Robert's ordtest to test the randomness of

# the data. Only useful when all of the community categories have been

# provided.

ot **<-** ordtest**(**p**$**species, community_categories**$**category, nitr**=**10000**)**

message**(**cat**(**"ordtest pvalue:", ot**$**p, sep**=**' '**))**

# Finish the script.

q **()**

**Section III: Controls for light reduction experiments**

Samples from 1996 were analyzed to determine if PE relative abundances changed over time at undisturbed control sites, and to replicate the effects of UV blocking on *Synechococcus* mat populations.

**Methods.** A 58.6 to 62.2°C site in the microbial mat of Mushroom Spring was covered with a wire mesh screen supported by a wooden platform placed ~1 to 2 cm above the water surface to protect the experimental area from possible hail damage. Samples were collected in duplicate by David Ward from 21 to 26 July 1996 using a #4 cork borer (38.5 mm^2^) every day between 1200 and 1800 h. Samples were immediately frozen and stored at -80°C until analysis in 2012. Molecular methods were the same as described in the main-text.

**Results and discussion.** PE diversity of the undisturbed experimental site mostly matched the diversity observed in at this temperature, where B′-like PEs are the predominant populations. In the unaltered control samples there was minimal change in the relative abundances of predominant PEs over a 5 day period (P >0.05; Supplementary Figure 3 and Supplementary Table 2).

**Supplementary References**

Jordan, P., Fromme, P., Witt, H.T., Klukas, O., Saenger, W., Krauss, N. (2001) Three-dimensional structure of cyanobacterial photosystem I at 2.5 Å resolution. *Nature* 411: 909-917.

**Supplementary Table 1.** Summary of G-test statistical analyses for flow path and vertical distributions, and PE-specific transcript studies. Statistics for main-text distributions are in bold. Letters in paraentheses correspond to Supplementary Figure 3 (vertical) and 5 (transcription).

| **Distribution** | **Number of samples analyzed** | **Number of ecotypes analyzed** | **Degrees of freedom** | **chi square value** | **P-value** |
| --- | --- | --- | --- | --- | --- |
| **flow path A/B′** | **4** | **11** | **30** | **1.10E+04** | **<0.001** |
| **flow path A** | **4** | **6** | **15** | **4.20E+03** | **<0.001** |
| **flow path B′** | **4** | **5** | **12** | **4.99E+02** | **<0.001** |
| **vertical 63°C A/B′** | **10** | **7** | **54** | **4.30E+03** | **<0.001** |
| **vertical 63°C A** | **10** | **4** | **27** | **6.72E+02** | **<0.001** |
| **vertical 63°C B′** | **10** | **3** | **18** | **1.30E+02** | **<0.001** |
| vertical 60°C A/B′ (3A) | 5 | 6 | 20 | 6.71E+02 | <0.001 |
| vertical 60°C A (3A) | 5 | 3 | 8 | 1.23E+02 | <0.001 |
| vertical 60°C B′ (3A) | 5 | 3 | 8 | 1.04E+02 | <0.001 |
| vertical 60°C A/B′ (3B) | 10 | 6 | 45 | 1.50E+03 | <0.001 |
| vertical 60°C A (3B) | 10 | 3 | 18 | 1.13E+02 | <0.001 |
| vertical 60°C B′ (3B) | 10 | 3 | 18 | 8.13E+02 | <0.001 |
| vertical 63°C A/B′ (3C) | 10 | 4 | 27 | 2.00E+03 | <0.001 |
| vertical 63°C A (3C) | 10 | 2 | 18 | 5.97E+01 | <0.001 |
| vertical 63°C B′ (3C) | 10 | 2 | 18 | 9.56E+02 | <0.001 |
| vertical 65°C A/B′ (3D) | 12 | 6 | 55 | 6.90E+03 | <0.001 |
| vertical 65°C A (3D) | 12 | 4 | 33 | 2.90E+03 | <0.001 |
| vertical 65°C B′ (3D) | 12 | 2 | 11 | 1.46E+02 | <0.001 |
| vertical 65°C A (3E) | 9 | 6 | 40 | 7.82E+02 | <0.001 |
| **transcription 60°C** | **18** | **4** | **48** | **6.93E+02** | **<0.001** |
| transcription 63°C (5A) | 4 | 3 | 6 | 1.69E+02 | <0.001 |
| transcription 65°C (5B) | 5 | 3 | 8 | 0.89E+02 | <0.001 |
| transcription 68°C (5C) | 4 | 2 | 3 | 0.31E+02 | <0.001 |

Supplementary Table 2. Summary of anova statistical analyses for perturbation studies and controls.

| **Experiment (year)** | **P-value** | **Fixation indices** | **Degrees of freedom** |
| --- | --- | --- | --- |
| Control (1996) | 0.35 | 1.15 | 2,24 |
| Light reduction (2008) | 0.026 | 4.40 | 3,12 |
| Temperature shift (2008) | 0.016 | 3.83 | 5,18 |

**Supplementary Table 3.** p–values associated with the hypothesis that the ratio of the number of singleton and low-frequency multi-sequence variants (LFSs) to the number of HFSs in predominant PE clades were different over the four day light reduction experiment (A) and temperature shift experiment (B).

**(A**)

| **PE** | **p-value** |
| --- | --- |
| **Bʹ9** | 0.5062 |
| **A1** | 0.5236 |
| **A6** | 0.5453 |
| **A14** | 0.9529 |

**(B**)

| **PE** | **p-value** |
| --- | --- |
| **A1** | 0.3423 |
| **A4** | 0.7679 |
| **A6** | 0.8335 |

**Supplementary Table 4. PE population percentages along replicate effluent flow path samples.**

|  | |  | **PE % in single temperature samples** | | | | | | | | | | |
| --- | --- | --- | --- | --- | --- | --- | --- | --- | --- | --- | --- | --- | --- |
| **PE** | | | | **Replicate sample 1** | | | | |  | **Replicate sample 2** | | | |
|  |  | | | **60°C** | **63°C** | **65°C** | **68°C** |  | **60°C** | | **63°C** | **65°C** | **68°C** |
| **A1** |  | | | 0.8 | 1.5 | 22.5 | 7.3 |  | 4.3 | | 4.2 | 31.0 | 2.0 |
| **A2** |  | | | 0.3 | 0.0 | 0.2 | 3.6 |  | 0.0 | | 0.6 | 0.5 | 0.3 |
| **A3** |  | | | 0.1 | 0.1 | 0.6 | 0.0 |  | 0.1 | | 0.0 | 0.3 | 0.0 |
| **A4** |  | | | 0.1 | 0.4 | 5.2 | 0.8 |  | 5.2 | | 0.5 | 2.0 | 0.3 |
| **A5** |  | | | 0.2 | 0.4 | 4.6 | 1.0 |  | 2.7 | | 1.6 | 11.6 | 0.2 |
| **A6** |  | | | 0.3 | 1.9 | 16.2 | 2.9 |  | 12.5 | | 4.5 | 10.2 | 0.9 |
| **A7** |  | | | 0.0 | 0.1 | 0.1 | 4.9 |  | 0.0 | | 0.2 | 8.9 | 1.4 |
| **A'8** |  | | | 0.0 | 0.0 | 0.0 | 7.9 |  | 0.0 | | 0.0 | 0.0 | 13.4 |
| **A'9** |  | | | 0.0 | 0.0 | 0.2 | 17.0 |  | 0.0 | | 0.0 | 0.3 | 29.1 |
| **A10** |  | | | 0.2 | 0.0 | 0.9 | 0.8 |  | 0.4 | | 0.3 | 0.6 | 0.1 |
| **A11** |  | | | 0.0 | 0.0 | 2.2 | 0.1 |  | 0.2 | | 0.1 | 0.5 | 0.0 |
| **A12** |  | | | 0.0 | 0.5 | 4.0 | 2.5 |  | 0.1 | | 0.1 | 21.2 | 1.1 |
| **A13** |  | | | 0.0 | 0.0 | 0.0 | 0.0 |  | 0.0 | | 0.0 | 0.2 | 0.3 |
| **A14** |  | | | 0.1 | 0.5 | 12.6 | 1.1 |  | 3.8 | | 2.0 | 8.7 | 0.0 |
| **A15** |  | | | 0.0 | 0.0 | 0.7 | 0.1 |  | 0.1 | | 0.0 | 0.6 | 0.0 |
| **A'16** |  | | | 0.0 | 0.0 | 0.1 | 6.2 |  | 0.0 | | 0.1 | 0.3 | 10.4 |
| **A'17** |  | | | 0.0 | 0.0 | 0.0 | 10.3 |  | 0.0 | | 0.0 | 0.0 | 18.9 |
| **A'18** |  | | | 0.0 | 0.0 | 0.0 | 0.4 |  | 0.0 | | 0.0 | 2.5 | 0.3 |
| **A19** |  | | | 0.0 | 0.0 | 0.0 | 3.7 |  | 0.0 | | 0.0 | 0.0 | 5.9 |
| **A20** |  | | | 0.0 | 0.0 | 0.1 | 1.4 |  | 0.0 | | 0.1 | 0.1 | 2.2 |
| **A'21** |  | | | 0.0 | 0.0 | 0.2 | 6.4 |  | 0.0 | | 0.1 | 0.2 | 9.4 |
| **A'22** |  | | | 0.0 | 0.1 | 0.0 | 0.1 |  | 0.0 | | 0.0 | 0.0 | 0.1 |
| **B'1** |  | | | 0.5 | 0.3 | 0.0 | 0.4 |  | 0.3 | | 0.2 | 0.0 | 0.3 |
| **B'2** |  | | | 21.3 | 13.5 | 2.6 | 1.0 |  | 10.0 | | 5.8 | 0.1 | 0.0 |
| **B'3** |  | | | 0.0 | 0.3 | 0.0 | 1.7 |  | 0.2 | | 0.3 | 0.0 | 0.0 |
| **B'4** |  | | | 0.0 | 0.0 | 0.0 | 0.0 |  | 0.0 | | 0.3 | 0.0 | 0.0 |
| **B'5** |  | | | 0.0 | 0.3 | 0.1 | 0.0 |  | 0.0 | | 0.2 | 0.0 | 0.0 |
| **B'6** |  | | | 0.1 | 0.0 | 0.0 | 1.4 |  | 0.0 | | 0.0 | 0.0 | 0.1 |
| **B'7** |  | | | 1.1 | 7.0 | 0.1 | 0.3 |  | 0.4 | | 3.3 | 0.0 | 0.1 |
| **B'8** |  | | | 8.8 | 2.2 | 2.6 | 1.4 |  | 8.3 | | 2.7 | 0.6 | 0.5 |
| **B'9** |  | | | 23.5 | 29.0 | 6.1 | 4.7 |  | 23.4 | | 26.8 | 0.7 | 1.6 |
| **B'10** |  | | | 0.0 | 0.0 | 0.3 | 0.0 |  | 0.3 | | 0.0 | 0.0 | 0.0 |
| **B'11** |  | | | 0.6 | 0.6 | 0.2 | 0.0 |  | 0.6 | | 1.3 | 0.0 | 0.1 |
| **B'12** |  | | | 5.9 | 9.3 | 2.0 | 1.6 |  | 3.6 | | 6.2 | 0.0 | 0.6 |
| **B'13** |  | | | 0.1 | 0.1 | 0.0 | 0.1 |  | 0.1 | | 0.2 | 0.0 | 0.0 |
| **B'14** |  | | | 0.0 | 1.5 | 0.0 | 0.0 |  | 0.0 | | 0.5 | 0.0 | 0.1 |
| **B'15** |  | | | 0.0 | 0.4 | 6.8 | 1.2 |  | 0.4 | | 0.1 | 3.3 | 0.8 |
| **B'16** |  | | | 0.0 | 0.7 | 0.0 | 0.0 |  | 0.0 | | 0.2 | 0.0 | 0.0 |
| **B'17** |  | | | 0.8 | 0.2 | 0.0 | 0.0 |  | 0.9 | | 1.3 | 0.0 | 0.0 |
| **B'18** |  | | | 0.9 | 0.8 | 0.3 | 0.1 |  | 0.7 | | 0.5 | 0.0 | 0.0 |
| **B'19** |  | | | 0.1 | 0.8 | 0.0 | 0.0 |  | 0.2 | | 0.5 | 0.0 | 0.0 |
| **B'20** |  | | | 0.0 | 0.0 | 0.0 | 0.0 |  | 0.0 | | 0.4 | 0.0 | 0.0 |
| **B'21** |  | | | 0.0 | 0.2 | 0.0 | 0.0 |  | 0.0 | | 0.0 | 0.0 | 0.0 |
| **B'22** |  | | | 0.0 | 0.0 | 0.0 | 0.0 |  | 0.0 | | 0.0 | 0.0 | 0.1 |
| **B'23** |  | | | 0.1 | 0.7 | 0.0 | 0.0 |  | 0.2 | | 0.5 | 0.0 | 0.0 |
| **B'24** |  | | | 0.0 | 0.0 | 0.2 | 0.1 |  | 0.1 | | 0.0 | 0.0 | 0.0 |

**Supplementary Table 5.** Percent population of predominant A-like, A′-like and Bʹ-like PEs in pooled vertical samples in relation to whole temperature samples.

|  | **60°C whole** | **60°C whole** | **MS60** | **MS60** | **63°C whole** | **63°C whole** | **MS63** | **MS63** | **65°C whole** | **65°C whole** | **MS65** | **MS65** | **68°C whole** | **68°C whole** |
| --- | --- | --- | --- | --- | --- | --- | --- | --- | --- | --- | --- | --- | --- | --- |
|  |  |  | **Vertical** | **Vertical** |  |  | **Vertical** | **Vertical** |  |  | **Vertical** | **Vertical** |  |  |
|  |  |  | **core 1** | **core 2** |  |  | **core 1** | **core 2** |  |  | **core 1** | **core 2** |  |  |
| **A1** | 0.8 | 4.3 | 6.6 | 6.7 | 1.5 | 4.2 | 6.7 | 15.9 | 22.5 | 31 | 14.4 | 29.1 | 7.3 | 2 |
| **A4** | 0.1 | 5.2 | 2.6 | 1.5 | 0.4 | 0.5 | 1.5 | 7.8 | 5.2 | 2 | 4.6 | 2.8 | 0.8 | 0.3 |
| **A5** | 0.2 | 2.7 | 1.2 | 0.9 | 0.4 | 1.6 | 0.9 | 2.9 | 4.6 | 11.6 | 2.4 | 5.2 | 1 | 0.2 |
| **A6** | 0.3 | 12.5 | 6.4 | 2.9 | 1.9 | 4.5 | 2.9 | 9.3 | 16.2 | 10.2 | 15.2 | 12.5 | 2.9 | 0.9 |
| **A7** | 0 | 0 | 0.2 | 0.1 | 0.1 | 0.2 | 0.1 | 1.6 | 0.1 | 8.9 | 0.4 | 10.9 | 4.9 | 1.4 |
| **Aʹ8** | 0 | 0 | 0 | 0 | 0 | 0 | 0 | 0 | 0 | 0 | 0 | 0 | 7.9 | 13.4 |
| **Aʹ9** | 0 | 0 | 0 | 0.1 | 0 | 0 | 0.1 | 0.2 | 0.2 | 0.3 | 0.2 | 0.4 | 17 | 29.1 |
| **A12** | 0 | 0.1 | 0.1 | 0.2 | 0.5 | 0.1 | 0.2 | 0.9 | 4 | 21.2 | 2 | 23.5 | 2.5 | 1.1 |
| **A14** | 0.1 | 3.8 | 4.8 | 4.2 | 0.5 | 2 | 4.2 | 5.3 | 12.6 | 8.7 | 16.3 | 5.5 | 1.1 | 0 |
| **Aʹ16** | 0 | 0 | 0 | 0.1 | 0 | 0.1 | 0.1 | 0 | 0.1 | 0.3 | 0.1 | 0.3 | 6.2 | 10.4 |
| **Aʹ17** | 0 | 0 | 0 | 0 | 0 | 0 | 0 | 0 | 0 | 0 | 0.2 | 0.4 | 10.3 | 18.9 |
| **Bʹ2** | 21.3 | 10 | 14.4 | 25.8 | 13.5 | 5.8 | 25.8 | 1.8 | 2.6 | 0.1 | 3.6 | 0 | 1 | 0 |
| **Bʹ7** | 1.1 | 0.4 | 0.8 | 0.7 | 7 | 3.3 | 0.7 | 3.8 | 0.1 | 0 | 0.4 | 0 | 0.3 | 0.1 |
| **Bʹ8** | 8.8 | 8.3 | 6.8 | 9.2 | 2.2 | 2.7 | 9.2 | 3.1 | 2.6 | 0.6 | 2.2 | 0.6 | 1.4 | 0.5 |
| **Bʹ9** | 23.5 | 23.4 | 23.9 | 21 | 29 | 26.8 | 21 | 16.2 | 6.1 | 0.7 | 11.6 | 2.3 | 4.7 | 1.6 |
| **Bʹ12** | 5.9 | 3.6 | 3.1 | 4.9 | 9.3 | 6.2 | 4.9 | 2.1 | 2 | 0 | 3 | 0.4 | 1.6 | 0.6 |
| **Bʹ15** | 0 | 0.4 | 0.1 | 0.3 | 0.4 | 0.1 | 0.3 | 7 | 6.8 | 3.3 | 1.7 | 1.4 | 1.2 | 0.8 |

**Supplementary Table 6.** PE population percentages along the vertical gradient at the ~60°C average midday temperature site.

|  | **% PE along 80 µm depth intervals** | | | | | | | | | |
| --- | --- | --- | --- | --- | --- | --- | --- | --- | --- | --- |
| **PE** | **0/80 µm** | **80/160 µm** | **160/240 µm** | **240/320 µm** | **320/400 µm** | **400/480 µm** | **480/560 µm** | **560/640 µm** | **640/720 µm** | **720/800 µm** |
| **A1** | 1.8 | 3.3 | 6.0 | 6.8 | 6.0 | 7.7 | 8.9 | 9.3 | 8.9 | 1.8 |
| **A2** | 0.0 | 0.1 | 0.4 | 0.0 | 0.6 | 0.2 | 0.1 | 0.0 | 0.2 | 0.0 |
| **A3** | 0.2 | 0.1 | 0.0 | 0.0 | 0.0 | 0.0 | 0.1 | 0.1 | 0.2 | 0.2 |
| **A4** | 0.6 | 0.4 | 1.7 | 1.7 | 2.3 | 1.8 | 1.5 | 2.2 | 1.9 | 0.6 |
| **A5** | 0.0 | 0.5 | 1.0 | 0.3 | 1.4 | 0.9 | 1.1 | 1.7 | 1.4 | 0.0 |
| **A6** | 0.2 | 0.4 | 2.0 | 1.4 | 2.6 | 3.7 | 4.1 | 3.7 | 6.7 | 0.2 |
| **A7** | 0.0 | 0.1 | 0.0 | 0.1 | 0.0 | 0.0 | 0.0 | 0.0 | 0.0 | 0.0 |
| **A'8** | 0.0 | 0.0 | 0.0 | 0.0 | 0.0 | 0.0 | 0.0 | 0.0 | 0.0 | 0.0 |
| **A'9** | 0.0 | 0.1 | 0.0 | 0.1 | 0.2 | 0.1 | 0.1 | 0.1 | 0.0 | 0.0 |
| **A10** | 0.1 | 0.0 | 0.5 | 0.0 | 0.0 | 0.1 | 0.0 | 0.0 | 0.2 | 0.1 |
| **A11** | 0.0 | 0.0 | 0.0 | 0.0 | 0.0 | 0.0 | 0.0 | 0.0 | 0.0 | 0.0 |
| **A12** | 0.0 | 0.0 | 0.9 | 0.0 | 0.1 | 0.1 | 0.0 | 0.4 | 0.0 | 0.0 |
| **A13** | 0.0 | 0.0 | 0.0 | 0.1 | 0.0 | 0.1 | 0.0 | 0.0 | 0.0 | 0.0 |
| **A14** | 0.3 | 1.6 | 2.5 | 2.3 | 4.4 | 4.1 | 3.7 | 9.7 | 6.6 | 0.3 |
| **A15** | 0.0 | 0.1 | 0.1 | 0.0 | 0.1 | 0.1 | 0.1 | 0.0 | 0.1 | 0.0 |
| **A'16** | 0.0 | 0.0 | 0.0 | 0.0 | 0.0 | 0.0 | 0.1 | 0.1 | 0.5 | 0.0 |
| **A'17** | 0.0 | 0.0 | 0.0 | 0.0 | 0.0 | 0.0 | 0.0 | 0.0 | 0.0 | 0.0 |
| **A'18** | 0.0 | 0.0 | 0.0 | 0.1 | 0.0 | 0.0 | 0.0 | 0.0 | 0.0 | 0.0 |
| **A19** | 0.0 | 0.0 | 0.0 | 0.0 | 0.0 | 0.0 | 0.0 | 0.0 | 0.0 | 0.0 |
| **A20** | 0.0 | 0.0 | 0.0 | 0.0 | 0.0 | 0.0 | 0.0 | 0.1 | 0.5 | 0.0 |
| **A'21** | 0.0 | 0.0 | 0.0 | 0.0 | 0.0 | 0.0 | 0.0 | 0.0 | 0.0 | 0.0 |
| **A'22** | 0.0 | 0.0 | 0.0 | 0.0 | 0.0 | 0.0 | 0.0 | 0.0 | 0.0 | 0.0 |
| **B'1** | 0.2 | 0.1 | 0.4 | 0.0 | 0.5 | 1.0 | 0.3 | 0.1 | 0.2 | 0.2 |
| **B'2** | 18.0 | 20.8 | 23.7 | 21.6 | 21.2 | 23.3 | 27.2 | 31.8 | 33.7 | 18.0 |
| **B'3** | 0.6 | 2.2 | 0.5 | 0.9 | 1.1 | 0.7 | 0.6 | 0.2 | 0.4 | 0.6 |
| **B'4** | 0.1 | 0.1 | 0.0 | 0.1 | 0.0 | 0.0 | 0.0 | 0.1 | 0.0 | 0.1 |
| **B'5** | 0.0 | 0.0 | 0.0 | 0.0 | 0.0 | 0.0 | 0.0 | 0.0 | 0.0 | 0.0 |
| **B'6** | 0.0 | 0.0 | 0.0 | 0.0 | 0.0 | 0.0 | 0.0 | 0.0 | 0.0 | 0.0 |
| **B'7** | 1.6 | 1.1 | 0.5 | 0.9 | 0.2 | 0.7 | 0.7 | 0.3 | 0.4 | 1.6 |
| **B'8** | 8.1 | 11.6 | 12.2 | 12.2 | 9.2 | 12.7 | 10.3 | 6.8 | 5.2 | 8.1 |
| **B'9** | 37.8 | 29.2 | 20.5 | 24.5 | 25.0 | 17.7 | 16.9 | 9.3 | 11.9 | 37.8 |
| **B'10** | 0.0 | 0.0 | 0.0 | 0.0 | 0.4 | 0.0 | 0.0 | 0.0 | 0.0 | 0.0 |
| **B'11** | 1.4 | 0.8 | 0.8 | 0.4 | 0.7 | 2.6 | 0.3 | 0.1 | 0.3 | 1.4 |
| **B'12** | 8.9 | 8.4 | 5.8 | 5.7 | 4.1 | 3.5 | 1.9 | 2.2 | 3.0 | 8.9 |
| **B'13** | 0.4 | 0.2 | 0.2 | 0.2 | 0.1 | 0.0 | 0.3 | 0.1 | 0.1 | 0.4 |
| **B'14** | 0.0 | 0.0 | 0.0 | 0.0 | 0.0 | 0.0 | 0.3 | 0.1 | 0.0 | 0.0 |
| **B'15** | 0.2 | 0.6 | 0.3 | 0.7 | 0.3 | 0.2 | 0.0 | 0.0 | 0.0 | 0.2 |
| **B'16** | 0.1 | 0.0 | 0.1 | 0.0 | 0.0 | 0.0 | 0.0 | 0.0 | 0.0 | 0.1 |
| **B'17** | 0.1 | 0.1 | 0.1 | 0.3 | 0.2 | 0.2 | 0.3 | 0.4 | 0.5 | 0.1 |
| **B'18** | 2.2 | 1.5 | 1.0 | 1.0 | 1.1 | 1.3 | 1.5 | 0.5 | 0.5 | 2.2 |
| **B'19** | 0.4 | 0.1 | 0.0 | 0.1 | 0.0 | 0.0 | 0.0 | 0.0 | 0.0 | 0.4 |
| **B'20** | 0.0 | 0.0 | 0.0 | 0.0 | 0.0 | 0.0 | 0.0 | 0.0 | 0.0 | 0.0 |
| **B'21** | 0.4 | 0.3 | 0.3 | 0.0 | 0.1 | 0.1 | 0.8 | 0.3 | 0.2 | 0.4 |
| **B'22** | 0.0 | 0.1 | 0.4 | 0.3 | 0.1 | 0.2 | 0.1 | 0.1 | 0.1 | 0.0 |
| **B'23** | 0.2 | 0.2 | 0.1 | 0.0 | 0.0 | 0.0 | 0.0 | 0.0 | 0.0 | 0.2 |
| **B'24** | 0.0 | 0.0 | 0.1 | 0.0 | 0.0 | 0.0 | 0.0 | 0.0 | 0.0 | 0.0 |

**Supplementary Table 7.** PE population percentages along the vertical gradient at ~60°C average midday temperature site.

|  | **% PE along 80 µm depth intervals Depth interval** | | | | |
| --- | --- | --- | --- | --- | --- |
| **PE** | **0/80 µm** | **80/160 µm** | **160/240 µm** | **240/320 µm** | **320/400 µm** |
| **A1** | 4.6 | 7.4 | 6.7 | 7.8 | 7.0 |
| **A2** | 0.2 | 0.1 | 0.1 | 0.1 | 0.2 |
| **A3** | 0.1 | 0.1 | 0.0 | 0.0 | 0.0 |
| **A4** | 0.8 | 2.1 | 3.5 | 4.7 | 3.0 |
| **A5** | 0.8 | 1.4 | 0.7 | 0.8 | 3.4 |
| **A6** | 0.7 | 4.1 | 7.1 | 11.9 | 13.6 |
| **A7** | 0.2 | 0.1 | 0.3 | 0.2 | 0.1 |
| **A'8** | 0.0 | 0.0 | 0.0 | 0.0 | 0.1 |
| **A'9** | 0.0 | 0.0 | 0.0 | 0.0 | 0.4 |
| **A10** | 0.1 | 0.2 | 0.5 | 0.7 | 0.6 |
| **A11** | 0.1 | 0.8 | 1.5 | 1.8 | 4.5 |
| **A12** | 0.2 | 0.0 | 0.0 | 0.0 | 0.4 |
| **A13** | 0.1 | 0.0 | 0.1 | 0.1 | 0.1 |
| **A14** | 0.9 | 3.2 | 5.8 | 8.6 | 9.4 |
| **A15** | 0.0 | 0.1 | 0.5 | 0.6 | 0.0 |
| **A'16** | 0.0 | 0.0 | 0.0 | 0.0 | 0.2 |
| **A'17** | 0.0 | 0.0 | 0.0 | 0.0 | 0.0 |
| **A'18** | 0.0 | 0.0 | 0.0 | 0.2 | 0.0 |
| **A19** | 0.0 | 0.0 | 0.0 | 0.0 | 0.0 |
| **A20** | 0.0 | 0.0 | 0.0 | 0.0 | 0.0 |
| **A'21** | 0.0 | 0.0 | 0.0 | 0.0 | 0.0 |
| **A'22** | 0.0 | 0.0 | 0.0 | 0.0 | 0.1 |
| **B'1** | 0.3 | 0.3 | 0.5 | 0.2 | 0.2 |
| **B'2** | 13.4 | 15.0 | 15.1 | 15.9 | 11.9 |
| **B'3** | 2.0 | 1.5 | 0.9 | 1.4 | 0.7 |
| **B'4** | 0.1 | 0.0 | 0.0 | 0.0 | 0.0 |
| **B'5** | 0.0 | 0.1 | 0.0 | 0.0 | 0.0 |
| **B'6** | 0.0 | 0.0 | 0.0 | 0.0 | 0.2 |
| **B'7** | 1.2 | 0.4 | 0.5 | 1.1 | 0.8 |
| **B'8** | 7.6 | 7.6 | 6.4 | 6.7 | 3.9 |
| **B'9** | 37.0 | 24.6 | 20.5 | 14.2 | 15.0 |
| **B'10** | 0.1 | 0.0 | 0.3 | 0.4 | 0.6 |
| **B'11** | 1.0 | 0.3 | 0.6 | 0.7 | 0.0 |
| **B'12** | 3.9 | 3.6 | 3.3 | 2.1 | 1.8 |
| **B'13** | 2.1 | 1.3 | 1.5 | 0.7 | 0.1 |
| **B'14** | 0.1 | 0.0 | 0.0 | 0.0 | 0.0 |
| **B'15** | 0.0 | 0.2 | 0.0 | 0.0 | 0.1 |
| **B'16** | 0.0 | 0.0 | 0.0 | 0.0 | 0.0 |
| **B'17** | 0.2 | 0.5 | 0.3 | 0.2 | 0.4 |
| **B'18** | 2.0 | 1.1 | 1.8 | 0.5 | 0.1 |
| **B'19** | 0.0 | 0.5 | 0.1 | 0.1 | 1.1 |
| **B'20** | 0.0 | 0.3 | 0.0 | 0.5 | 0.0 |
| **B'21** | 0.1 | 0.1 | 0.0 | 0.0 | 0.0 |
| **B'22** | 0.1 | 0.0 | 0.0 | 0.2 | 0.0 |
| **B'23** | 0.0 | 0.0 | 0.0 | 0.2 | 0.0 |
| **B'24** | 0.0 | 0.0 | 0.0 | 0.0 | 0.0 |

**Supplementary Table 8.** PE population percentages along the vertical gradient at ~63°C average midday temperature site.

|  | **% PE along 80 µm depth intervals Depth interval** | | | | | | | | | | |
| --- | --- | --- | --- | --- | --- | --- | --- | --- | --- | --- | --- |
| **PE** | **0/80 µm** | **80/160 µm** | **160/240 µm** | **240/320 µm** | **320/400 µm** | **400/480 µm** | **480/560 µm** | **560/640 µm** | **640/720 µm** | **720/800 µm** | **800/880 µm** |
| **A1** | 10.8 | 13.5 | 0.8 | 4.5 | 8.2 | 4.7 | 8.5 | 1.8 | 8.4 | 10.8 | 1.8 |
| **A2** | 0.2 | 0.3 | 0.1 | 0.0 | 0.2 | 0.3 | 0.2 | 0.0 | 0.0 | 0.0 | 0.0 |
| **A3** | 0.1 | 0.2 | 0.2 | 0.6 | 0.0 | 0.0 | 0.0 | 0.0 | 0.0 | 0.0 | 0.0 |
| **A4** | 2.2 | 1.4 | 0.4 | 0.3 | 0.9 | 2.2 | 2.1 | 1.1 | 3.2 | 2.9 | 0.6 |
| **A5** | 2.1 | 4.1 | 0.2 | 1.0 | 0.8 | 1.1 | 2.6 | 0.4 | 1.4 | 2.1 | 0.0 |
| **A6** | 2.8 | 2.5 | 0.0 | 0.0 | 0.2 | 1.4 | 2.4 | 1.1 | 1.7 | 2.4 | 1.1 |
| **A7** | 0.0 | 2.3 | 0.0 | 0.2 | 0.0 | 0.8 | 0.3 | 0.1 | 0.1 | 0.0 | 0.1 |
| **A'8** | 0.0 | 0.1 | 0.0 | 0.0 | 0.0 | 0.1 | 0.0 | 0.0 | 0.0 | 0.0 | 0.0 |
| **A'9** | 0.0 | 0.0 | 0.0 | 0.0 | 0.3 | 0.3 | 0.0 | 0.1 | 0.0 | 0.0 | 0.0 |
| **A10** | 0.1 | 0.8 | 0.0 | 0.0 | 0.3 | 0.0 | 0.0 | 0.0 | 0.6 | 0.2 | 0.0 |
| **A11** | 0.1 | 0.4 | 0.0 | 0.0 | 0.0 | 0.1 | 0.1 | 0.0 | 0.1 | 0.0 | 0.0 |
| **A12** | 0.0 | 1.4 | 0.0 | 0.4 | 0.2 | 0.5 | 0.9 | 0.0 | 0.0 | 0.3 | 0.0 |
| **A13** | 0.0 | 0.0 | 0.0 | 0.0 | 0.1 | 0.1 | 0.1 | 0.1 | 0.0 | 0.0 | 0.0 |
| **A14** | 3.2 | 2.0 | 0.0 | 0.5 | 1.6 | 1.2 | 4.0 | 1.0 | 2.8 | 3.6 | 1.1 |
| **A15** | 0.1 | 0.2 | 0.0 | 0.0 | 0.1 | 0.1 | 0.0 | 0.0 | 0.1 | 0.1 | 0.0 |
| **A'16** | 0.0 | 0.1 | 0.0 | 0.1 | 0.0 | 0.0 | 0.0 | 0.0 | 0.1 | 0.0 | 0.0 |
| **A'17** | 0.0 | 0.1 | 0.0 | 0.0 | 0.0 | 0.0 | 0.0 | 0.0 | 0.0 | 0.3 | 0.0 |
| **A'18** | 0.0 | 0.7 | 0.0 | 0.0 | 0.0 | 0.0 | 0.0 | 0.0 | 0.0 | 0.0 | 0.0 |
| **A19** | 0.0 | 0.0 | 0.0 | 0.0 | 0.0 | 0.0 | 0.0 | 0.0 | 0.0 | 0.0 | 0.0 |
| **A20** | 0.0 | 0.0 | 0.0 | 0.0 | 0.0 | 0.0 | 0.0 | 0.0 | 0.0 | 0.0 | 0.0 |
| **A'21** | 0.0 | 0.0 | 0.0 | 0.5 | 0.0 | 0.0 | 0.0 | 0.0 | 0.0 | 0.0 | 0.0 |
| **A'22** | 0.0 | 0.2 | 0.0 | 0.0 | 0.0 | 0.0 | 0.0 | 0.0 | 0.0 | 0.0 | 0.1 |
| **B'1** | 0.0 | 0.1 | 0.0 | 0.0 | 0.4 | 0.0 | 0.0 | 0.0 | 0.0 | 0.0 | 0.0 |
| **B'2** | 8.9 | 3.2 | 4.6 | 4.9 | 10.5 | 7.5 | 16.4 | 6.6 | 13.9 | 8.8 | 4.8 |
| **B'3** | 0.8 | 1.5 | 0.1 | 0.9 | 1.1 | 1.6 | 0.3 | 1.7 | 1.3 | 0.7 | 0.7 |
| **B'4** | 0.0 | 0.0 | 0.0 | 0.0 | 0.0 | 0.0 | 0.0 | 0.1 | 0.0 | 0.2 | 0.0 |
| **B'5** | 0.1 | 0.1 | 0.0 | 0.0 | 0.0 | 0.2 | 0.1 | 0.0 | 0.1 | 0.0 | 0.0 |
| **B'6** | 0.0 | 0.2 | 0.0 | 0.0 | 0.0 | 0.2 | 0.1 | 0.0 | 0.0 | 0.0 | 0.0 |
| **B'7** | 2.1 | 2.9 | 5.8 | 3.6 | 2.1 | 2.6 | 1.3 | 3.5 | 2.2 | 2.5 | 11.2 |
| **B'8** | 4.8 | 2.0 | 0.4 | 1.2 | 5.0 | 5.8 | 5.1 | 2.4 | 4.7 | 5.8 | 0.8 |
| **B'9** | 23.7 | 40.2 | 72.8 | 55.3 | 35.7 | 26.1 | 13.2 | 48.9 | 18.9 | 22.8 | 50.6 |
| **B'10** | 0.3 | 0.8 | 0.0 | 0.0 | 0.4 | 0.3 | 0.0 | 0.2 | 0.0 | 0.0 | 0.0 |
| **B'11** | 0.4 | 0.6 | 1.3 | 1.0 | 1.0 | 0.5 | 0.2 | 0.7 | 0.5 | 0.7 | 1.1 |
| **B'12** | 3.6 | 5.0 | 4.7 | 8.7 | 3.5 | 4.4 | 2.4 | 7.6 | 1.8 | 3.3 | 7.3 |
| **B'13** | 1.1 | 0.0 | 0.0 | 0.2 | 2.4 | 2.8 | 1.5 | 5.6 | 0.8 | 0.6 | 4.5 |
| **B'14** | 0.1 | 0.0 | 0.1 | 0.0 | 0.1 | 0.7 | 0.3 | 0.3 | 0.2 | 0.1 | 1.4 |
| **B'15** | 1.1 | 1.2 | 0.0 | 0.8 | 0.2 | 0.5 | 0.3 | 0.6 | 0.1 | 0.4 | 0.3 |
| **B'16** | 0.1 | 0.7 | 0.8 | 1.3 | 0.3 | 0.1 | 0.2 | 0.3 | 0.4 | 0.3 | 0.5 |
| **B'17** | 0.4 | 0.1 | 0.2 | 0.3 | 1.7 | 0.8 | 0.6 | 0.2 | 0.7 | 0.9 | 0.2 |
| **B'18** | 1.2 | 0.6 | 2.6 | 2.7 | 1.3 | 0.5 | 0.6 | 0.4 | 1.3 | 1.0 | 0.1 |
| **B'19** | 0.0 | 0.0 | 0.0 | 0.5 | 0.1 | 0.1 | 0.0 | 0.2 | 0.0 | 0.1 | 1.1 |
| **B'20** | 0.0 | 0.0 | 0.0 | 0.2 | 0.0 | 0.1 | 0.0 | 0.0 | 0.0 | 0.0 | 0.1 |
| **B'21** | 0.0 | 0.1 | 0.0 | 0.1 | 0.0 | 0.0 | 0.0 | 0.0 | 0.0 | 0.0 | 0.3 |
| **B'22** | 0.1 | 0.0 | 0.0 | 0.6 | 0.1 | 0.1 | 0.2 | 0.0 | 0.0 | 0.0 | 0.0 |
| **B'23** | 0.2 | 0.2 | 0.4 | 0.3 | 0.0 | 0.0 | 0.0 | 0.0 | 0.1 | 0.2 | 0.3 |
| **B'24** | 0.0 | 0.1 | 0.0 | 0.0 | 0.0 | 0.2 | 0.0 | 0.0 | 0.1 | 0.1 | 0.0 |

**Supplementary Table 9. PE population percentages along the vertical gradient at the ~63°C average midday temperature site.**

|  | | **% PE along 80 µm depth intervals Depth interval** | | | | | | | | | | |
| --- | --- | --- | --- | --- | --- | --- | --- | --- | --- | --- | --- | --- |
| **PE** | **0/80 µm** | | **80/160 µm** | **160/240 µm** | **240/320 µm** | **320/400 µm** | **400/480 µm** | **480/560 µm** | | **560/640 µm** | **640/720 µm** | **720/800 µm** |
| **A1** | 7.7 | | 11.5 | 11.2 | 12.2 | 16.4 | 20.3 | 20.3 | 21.1 | | 19.5 | 16.7 |
| **A2** | 0.0 | | 0.0 | 0.2 | 0.2 | 0.3 | 0.2 | 0.2 | 0.7 | | 0.3 | 0.6 |
| **A3** | 4.0 | | 1.5 | 1.1 | 2.0 | 0.6 | 0.4 | 0.4 | 0.1 | | 0.0 | 0.3 |
| **A4** | 0.2 | | 5.2 | 3.3 | 8.1 | 7.7 | 8.9 | 8.1 | 8.8 | | 16.0 | 11.8 |
| **A5** | 1.2 | | 2.6 | 3.5 | 1.4 | 1.4 | 1.1 | 5.0 | 3.3 | | 3.0 | 7.2 |
| **A6** | 0.1 | | 4.3 | 3.1 | 5.6 | 5.8 | 5.9 | 7.2 | 12.1 | | 24.3 | 25.6 |
| **A7** | 0.3 | | 1.2 | 1.4 | 0.2 | 1.1 | 2.6 | 2.2 | 3.2 | | 1.8 | 1.5 |
| **A'8** | 0.0 | | 0.0 | 0.0 | 0.0 | 0.0 | 0.1 | 0.0 | 0.0 | | 0.0 | 0.0 |
| **A'9** | 0.5 | | 0.3 | 0.4 | 0.1 | 0.3 | 0.1 | 0.0 | 0.0 | | 0.1 | 0.0 |
| **A10** | 0.6 | | 0.6 | 0.8 | 0.5 | 0.4 | 0.2 | 0.1 | 0.0 | | 0.1 | 0.1 |
| **A11** | 0.0 | | 0.2 | 0.6 | 0.3 | 0.6 | 1.1 | 0.9 | 2.0 | | 4.4 | 5.2 |
| **A12** | 0.1 | | 0.7 | 1.2 | 1.7 | 1.2 | 0.7 | 0.9 | 1.0 | | 0.6 | 0.5 |
| **A13** | 0.0 | | 0.0 | 0.0 | 0.1 | 0.2 | 0.1 | 0.1 | 0.0 | | 0.1 | 0.3 |
| **A14** | 0.9 | | 3.2 | 2.4 | 3.0 | 3.0 | 4.3 | 4.5 | 7.4 | | 13.2 | 12.2 |
| **A15** | 0.0 | | 0.0 | 0.2 | 0.1 | 0.1 | 0.0 | 0.4 | 0.7 | | 0.1 | 0.1 |
| **A'16** | 0.1 | | 0.0 | 0.1 | 0.1 | 0.0 | 0.0 | 0.0 | 0.0 | | 0.1 | 0.0 |
| **A'17** | 0.0 | | 0.0 | 0.0 | 0.0 | 0.0 | 0.0 | 0.0 | 0.0 | | 0.0 | 0.0 |
| **A'18** | 0.2 | | 0.2 | 0.2 | 0.0 | 0.3 | 0.0 | 0.5 | 0.7 | | 0.3 | 0.3 |
| **A19** | 0.0 | | 0.0 | 0.0 | 0.0 | 0.0 | 0.0 | 0.0 | 0.0 | | 0.0 | 0.0 |
| **A20** | 0.1 | | 0.0 | 0.0 | 0.0 | 0.0 | 0.0 | 0.0 | 0.0 | | 0.1 | 0.0 |
| **A'21** | 0.0 | | 0.0 | 0.1 | 0.0 | 0.0 | 0.0 | 0.0 | 0.0 | | 0.0 | 0.0 |
| **A'22** | 1.8 | | 0.4 | 0.7 | 0.3 | 0.4 | 0.3 | 0.2 | 0.0 | | 0.0 | 0.0 |
| **B'1** | 0.1 | | 0.0 | 0.0 | 0.1 | 0.0 | 0.0 | 0.1 | 0.0 | | 0.0 | 0.2 |
| **B'2** | 4.5 | | 1.6 | 2.1 | 1.7 | 2.0 | 1.8 | 1.9 | 0.8 | | 0.5 | 0.8 |
| **B'3** | 0.2 | | 0.5 | 0.4 | 0.5 | 0.3 | 0.4 | 0.1 | 1.2 | | 0.4 | 0.2 |
| **B'4** | 0.0 | | 0.0 | 0.0 | 0.0 | 0.0 | 0.0 | 0.0 | 0.1 | | 0.0 | 0.0 |
| **B'5** | 0.1 | | 0.5 | 0.6 | 0.9 | 0.4 | 1.0 | 0.4 | 0.8 | | 0.4 | 0.4 |
| **B'6** | 0.0 | | 0.0 | 0.3 | 0.0 | 0.0 | 0.0 | 0.0 | 0.1 | | 0.0 | 0.0 |
| **B'7** | 11.3 | | 6.6 | 6.1 | 4.4 | 3.0 | 2.5 | 2.8 | 1.3 | | 0.3 | 0.1 |
| **B'8** | 1.6 | | 2.7 | 3.9 | 2.7 | 4.0 | 4.8 | 3.3 | 4.0 | | 1.1 | 1.9 |
| **B'9** | 35.0 | | 19.8 | 19.7 | 23.0 | 21.3 | 16.8 | 13.3 | 7.6 | | 2.1 | 2.3 |
| **B'10** | 0.1 | | 0.3 | 0.2 | 0.7 | 0.2 | 0.3 | 0.6 | 0.1 | | 0.1 | 0.0 |
| **B'11** | 0.9 | | 0.1 | 0.4 | 0.2 | 0.5 | 0.0 | 0.5 | 0.0 | | 0.0 | 0.0 |
| **B'12** | 7.1 | | 9.2 | 7.2 | 7.2 | 5.9 | 6.6 | 6.3 | 5.4 | | 1.9 | 2.5 |
| **B'13** | 0.0 | | 0.0 | 0.0 | 0.0 | 0.0 | 0.0 | 0.0 | 0.0 | | 0.0 | 0.0 |
| **B'14** | 0.2 | | 0.2 | 0.2 | 0.3 | 0.5 | 0.2 | 0.4 | 0.7 | | 0.2 | 0.2 |
| **B'15** | 10.4 | | 12.2 | 10.1 | 10.1 | 8.0 | 5.9 | 7.1 | 4.6 | | 1.2 | 0.9 |
| **B'16** | 0.4 | | 0.0 | 0.0 | 0.0 | 0.1 | 0.0 | 0.0 | 0.0 | | 0.0 | 0.0 |
| **B'17** | 0.3 | | 0.1 | 0.0 | 0.1 | 0.0 | 0.0 | 0.0 | 0.0 | | 0.2 | 0.1 |
| **B'18** | 0.7 | | 1.2 | 1.2 | 0.1 | 0.1 | 0.3 | 0.2 | 0.0 | | 0.0 | 0.0 |
| **B'19** | 0.0 | | 0.0 | 0.0 | 0.0 | 0.2 | 0.0 | 0.0 | 0.0 | | 0.0 | 0.0 |
| **B'20** | 0.0 | | 0.0 | 0.0 | 0.0 | 0.1 | 0.2 | 0.2 | 0.0 | | 0.0 | 0.0 |
| **B'21** | 0.0 | | 0.1 | 0.3 | 0.0 | 0.0 | 0.0 | 0.0 | 0.2 | | 0.0 | 0.0 |
| **B'22** | 0.3 | | 0.0 | 0.0 | 0.0 | 0.1 | 0.0 | 0.0 | 0.0 | | 0.0 | 0.1 |
| **B'23** | 0.0 | | 0.0 | 0.0 | 0.0 | 0.0 | 0.0 | 0.0 | 0.0 | | 0.0 | 0.0 |
| **B'24** | 0.1 | | 0.0 | 0.2 | 0.0 | 0.0 | 0.0 | 0.0 | 0.0 | | 0.0 | 0.0 |

**Supplementary Table 10.** PE population percentages along the vertical gradient at ~65°C average midday temperature site.

|  | | **% PE along 80 µm depth intervals Depth interval** | | | | | | | | | | | |
| --- | --- | --- | --- | --- | --- | --- | --- | --- | --- | --- | --- | --- | --- |
| **PE** | **0/80 µm** | | **80/160 µm** | **160/240 µm** | **240/320 µm** | **320/400 µm** | **400/480 µm** | **480/560 µm** | **560/640 µm** | **640/720 µm** | **720/800 µm** | **800/880 µm** | **880/960 µm** |
| **A1** | 12.4 | | 13.5 | 18.3 | 17.7 | 4.7 | 19.2 | 21.5 | 19.3 | 17.9 | 10.9 | 7.1 | 8.0 |
| **A2** | 6.0 | | 0.2 | 0.4 | 0.0 | 0.3 | 0.1 | 0.1 | 0.2 | 0.2 | 0.0 | 0.1 | 0.3 |
| **A3** | 0.2 | | 1.5 | 1.0 | 1.0 | 0.0 | 0.3 | 0.1 | 0.0 | 0.1 | 0.0 | 0.0 | 0.1 |
| **A4** | 1.1 | | 1.5 | 3.7 | 2.6 | 2.2 | 5.9 | 8.0 | 7.1 | 7.5 | 6.1 | 4.5 | 3.2 |
| **A5** | 3.8 | | 1.2 | 2.9 | 4.4 | 1.1 | 3.0 | 3.2 | 4.0 | 1.4 | 1.7 | 0.7 | 1.7 |
| **A6** | 6.3 | | 1.0 | 0.6 | 2.3 | 1.4 | 8.7 | 9.0 | 14.3 | 19.7 | 30.5 | 37.3 | 42.4 |
| **A7** | 4.9 | | 0.1 | 0.5 | 0.5 | 0.8 | 0.2 | 0.2 | 0.1 | 0.0 | 0.0 | 0.0 | 0.5 |
| **A'8** | 0.0 | | 0.0 | 0.0 | 0.0 | 0.1 | 0.0 | 0.0 | 0.0 | 0.0 | 0.0 | 0.0 | 0.0 |
| **A'9** | 0.4 | | 0.0 | 0.2 | 0.2 | 0.3 | 0.0 | 0.1 | 0.0 | 0.2 | 0.4 | 0.2 | 0.2 |
| **A10** | 2.5 | | 1.5 | 0.5 | 1.0 | 0.0 | 0.2 | 0.2 | 0.0 | 0.3 | 0.1 | 0.1 | 0.0 |
| **A11** | 0.2 | | 0.1 | 0.0 | 0.1 | 0.1 | 1.1 | 0.6 | 1.1 | 1.1 | 1.5 | 1.0 | 0.7 |
| **A12** | 3.3 | | 1.1 | 2.4 | 3.7 | 0.5 | 3.4 | 3.5 | 2.2 | 2.6 | 0.9 | 0.6 | 0.4 |
| **A13** | 0.0 | | 0.0 | 0.0 | 0.1 | 0.1 | 1.1 | 0.4 | 0.2 | 0.6 | 0.2 | 0.4 | 0.1 |
| **A14** | 6.9 | | 2.0 | 0.9 | 2.5 | 1.2 | 11.2 | 12.6 | 19.2 | 21.9 | 35.6 | 39.4 | 31.3 |
| **A15** | 0.2 | | 0.1 | 0.0 | 0.1 | 0.1 | 0.3 | 0.3 | 0.1 | 0.8 | 0.6 | 1.0 | 0.7 |
| **A'16** | 0.2 | | 0.0 | 0.3 | 0.1 | 0.0 | 0.1 | 0.0 | 0.0 | 0.1 | 0.3 | 0.1 | 0.2 |
| **A'17** | 0.0 | | 0.0 | 0.0 | 0.1 | 0.0 | 0.4 | 0.0 | 0.7 | 0.0 | 0.2 | 0.3 | 0.2 |
| **A'18** | 0.6 | | 0.0 | 0.0 | 0.2 | 0.0 | 0.1 | 0.0 | 0.0 | 0.0 | 0.0 | 0.0 | 0.2 |
| **A19** | 0.1 | | 0.0 | 0.0 | 0.0 | 0.0 | 0.0 | 0.1 | 0.0 | 0.0 | 0.1 | 0.2 | 0.0 |
| **A20** | 0.2 | | 0.0 | 0.1 | 0.1 | 0.0 | 0.0 | 0.0 | 0.0 | 0.0 | 0.2 | 0.0 | 0.2 |
| **A'21** | 0.0 | | 0.0 | 0.3 | 0.8 | 0.0 | 0.2 | 0.3 | 0.2 | 0.2 | 0.9 | 0.9 | 0.5 |
| **A'22** | 0.0 | | 2.3 | 0.0 | 0.5 | 0.0 | 0.5 | 0.0 | 0.0 | 0.0 | 0.0 | 0.4 | 0.0 |
| **B'1** | 0.0 | | 0.4 | 0.3 | 0.0 | 0.0 | 0.0 | 0.0 | 0.0 | 0.1 | 0.0 | 0.0 | 0.0 |
| **B'2** | 3.8 | | 8.2 | 3.1 | 3.8 | 7.5 | 3.7 | 3.1 | 3.6 | 2.7 | 1.7 | 0.6 | 1.7 |
| **B'3** | 1.9 | | 1.5 | 3.1 | 0.5 | 1.6 | 0.3 | 0.2 | 0.1 | 0.2 | 0.1 | 0.0 | 0.0 |
| **B'4** | 0.0 | | 0.0 | 0.0 | 0.0 | 0.0 | 0.0 | 0.0 | 0.0 | 0.0 | 0.0 | 0.0 | 0.0 |
| **B'5** | 0.0 | | 0.0 | 0.1 | 0.1 | 0.2 | 0.1 | 0.0 | 0.0 | 0.0 | 0.0 | 0.0 | 0.0 |
| **B'6** | 0.5 | | 0.1 | 0.1 | 0.0 | 0.2 | 0.0 | 0.0 | 0.0 | 0.0 | 0.0 | 0.0 | 0.0 |
| **B'7** | 1.2 | | 1.0 | 0.3 | 0.1 | 2.6 | 0.0 | 0.0 | 0.0 | 0.1 | 0.0 | 0.0 | 0.0 |
| **B'8** | 4.7 | | 3.6 | 1.6 | 1.6 | 5.8 | 2.6 | 2.1 | 1.3 | 1.7 | 1.4 | 0.5 | 0.9 |
| **B'9** | 13.5 | | 20.3 | 26.7 | 21.6 | 26.1 | 11.3 | 9.9 | 5.2 | 5.3 | 1.4 | 1.4 | 2.0 |
| **B'10** | 0.0 | | 0.2 | 0.0 | 0.3 | 0.3 | 0.2 | 0.0 | 0.6 | 0.0 | 0.0 | 0.0 | 0.2 |
| **B'11** | 0.7 | | 0.6 | 0.5 | 0.3 | 0.5 | 0.1 | 0.2 | 0.0 | 0.1 | 0.0 | 0.1 | 0.0 |
| **B'12** | 3.6 | | 9.1 | 7.2 | 4.8 | 4.4 | 1.6 | 1.4 | 1.5 | 1.7 | 1.1 | 0.5 | 0.5 |
| **B'13** | 0.0 | | 0.4 | 0.0 | 0.0 | 2.8 | 0.0 | 0.0 | 0.0 | 0.0 | 0.0 | 0.0 | 0.0 |
| **B'14** | 0.0 | | 0.2 | 0.0 | 0.1 | 0.7 | 0.0 | 0.2 | 0.0 | 0.0 | 0.0 | 0.0 | 0.0 |
| **B'15** | 4.7 | | 2.0 | 4.3 | 3.4 | 0.5 | 1.7 | 3.8 | 0.6 | 0.8 | 0.1 | 0.3 | 0.3 |
| **B'16** | 0.0 | | 0.0 | 0.0 | 0.0 | 0.1 | 0.0 | 0.0 | 0.0 | 0.0 | 0.0 | 0.0 | 0.0 |
| **B'17** | 0.0 | | 0.5 | 0.0 | 0.0 | 0.8 | 0.0 | 0.0 | 0.0 | 0.0 | 0.0 | 0.0 | 0.0 |
| **B'18** | 0.5 | | 1.0 | 0.8 | 0.7 | 0.5 | 0.2 | 0.2 | 0.4 | 0.0 | 0.1 | 0.1 | 0.0 |
| **B'19** | 0.0 | | 0.8 | 1.0 | 1.0 | 0.1 | 0.2 | 0.2 | 0.0 | 0.0 | 0.0 | 0.0 | 0.0 |
| **B'20** | 0.0 | | 0.0 | 0.0 | 0.0 | 0.1 | 0.0 | 0.0 | 0.0 | 0.0 | 0.0 | 0.0 | 0.0 |
| **B'21** | 0.0 | | 0.0 | 0.0 | 0.0 | 0.0 | 0.0 | 0.0 | 0.0 | 0.0 | 0.0 | 0.0 | 0.0 |
| **B'22** | 0.7 | | 0.0 | 0.1 | 0.0 | 0.1 | 0.1 | 0.0 | 0.0 | 0.0 | 0.0 | 0.1 | 0.1 |
| **B'23** | 0.0 | | 0.1 | 0.0 | 0.0 | 0.0 | 0.1 | 0.0 | 0.0 | 0.0 | 0.0 | 0.0 | 0.0 |
| **B'24** | 0.0 | | 0.2 | 0.1 | 0.0 | 0.2 | 0.1 | 0.0 | 0.2 | 0.2 | 0.0 | 0.1 | 0.0 |

**Supplementary Table 11. PE population percentages along the vertical gradient at the ~65°C average midday temperature site.**

|  | | **% PE along 80 µm depth intervals Depth interval** | | | | | | | | |
| --- | --- | --- | --- | --- | --- | --- | --- | --- | --- | --- |
|  | **0/80µm** | | **80/160µm** | **160/240µm** | **240/320µm** | **320/400µm** | **400/480µm** | **480/560µm** | **560/640µm** | **640/720µm** |
| **A1** | 35.4 | | 35.1 | 33.8 | 26.0 | 27.3 | 22.4 | 30.7 | 26.0 | 25.2 |
| **A2** | 0.4 | | 0.5 | 1.2 | 1.3 | 0.6 | 0.7 | 1.0 | 0.9 | 0.7 |
| **A3** | 0.9 | | 1.4 | 0.8 | 0.4 | 0.2 | 0.2 | 0.2 | 0.1 | 0.3 |
| **A4** | 0.5 | | 2.9 | 2.2 | 3.5 | 3.8 | 2.9 | 3.7 | 2.6 | 2.6 |
| **A5** | 7.1 | | 7.2 | 3.7 | 6.3 | 3.1 | 2.5 | 1.7 | 3.5 | 12.6 |
| **A6** | 4.2 | | 7.6 | 9.6 | 15.3 | 20.9 | 20.6 | 14.1 | 10.8 | 8.0 |
| **A7** | 14.4 | | 12.9 | 7.8 | 8.8 | 10.7 | 8.5 | 11.2 | 12.9 | 11.7 |
| **A'8** | 0.0 | | 0.0 | 0.0 | 0.0 | 0.0 | 0.0 | 0.0 | 0.0 | 0.0 |
| **A'9** | 0.0 | | 0.0 | 0.4 | 0.3 | 0.2 | 0.2 | 0.6 | 1.7 | 0.0 |
| **A10** | 1.2 | | 1.3 | 0.3 | 0.1 | 0.1 | 0.2 | 0.1 | 0.2 | 0.1 |
| **A11** | 0.1 | | 0.1 | 0.1 | 0.6 | 0.3 | 1.1 | 0.2 | 0.5 | 0.5 |
| **A12** | 24.2 | | 19.5 | 24.3 | 22.4 | 19.0 | 23.7 | 22.1 | 28.7 | 29.7 |
| **A13** | 0.4 | | 0.1 | 0.2 | 0.1 | 0.2 | 0.2 | 0.0 | 0.1 | 0.1 |
| **A14** | 2.6 | | 4.0 | 4.2 | 7.5 | 8.1 | 7.3 | 6.3 | 4.7 | 4.3 |
| **A15** | 0.4 | | 0.4 | 0.5 | 0.6 | 0.2 | 0.4 | 0.4 | 0.1 | 0.7 |
| **A'16** | 0.2 | | 0.1 | 0.2 | 0.3 | 0.2 | 0.4 | 0.5 | 0.6 | 0.3 |
| **A'17** | 0.2 | | 0.2 | 0.1 | 0.1 | 0.2 | 0.6 | 0.7 | 1.3 | 0.0 |
| **A'18** | 2.5 | | 2.6 | 0.9 | 1.6 | 1.3 | 1.0 | 0.7 | 2.1 | 3.2 |
| **A19** | 0.0 | | 0.0 | 0.0 | 0.0 | 0.0 | 0.2 | 0.0 | 0.2 | 0.0 |
| **A20** | 0.0 | | 0.1 | 0.2 | 0.1 | 0.0 | 0.0 | 0.3 | 0.2 | 0.3 |
| **A'21** | 0.2 | | 0.0 | 0.4 | 0.7 | 0.2 | 1.0 | 0.9 | 3.3 | 0.5 |
| **A'22** | 0.1 | | 0.2 | 0.0 | 0.0 | 0.0 | 0.0 | 0.0 | 0.0 | 0.0 |
| **B'1** | 0.1 | | 0.1 | 0.0 | 0.0 | 0.0 | 0.0 | 0.0 | 0.0 | 0.0 |
| **B'2** | 0.0 | | 0.1 | 0.1 | 0.0 | 0.0 | 0.0 | 0.0 | 0.0 | 0.0 |
| **B'3** | 0.0 | | 0.1 | 0.0 | 0.1 | 0.0 | 0.0 | 0.0 | 0.1 | 0.0 |
| **B'4** | 0.0 | | 0.0 | 0.0 | 0.0 | 0.0 | 0.0 | 0.0 | 0.0 | 0.0 |
| **B'5** | 0.0 | | 0.4 | 1.5 | 2.2 | 1.3 | 1.3 | 0.8 | 0.6 | 0.1 |
| **B'6** | 0.0 | | 0.0 | 0.0 | 0.0 | 0.0 | 0.0 | 0.0 | 0.0 | 0.0 |
| **B'7** | 0.0 | | 0.2 | 0.0 | 0.0 | 0.0 | 0.0 | 0.0 | 0.1 | 0.1 |
| **B'8** | 0.7 | | 0.5 | 0.4 | 0.8 | 0.2 | 0.7 | 0.8 | 0.2 | 0.7 |
| **B'9** | 2.9 | | 2.9 | 3.3 | 1.7 | 1.7 | 2.9 | 2.3 | 1.3 | 1.4 |
| **B'10** | 0.0 | | 0.0 | 0.1 | 0.1 | 0.0 | 0.0 | 0.0 | 0.0 | 0.0 |
| **B'11** | 0.0 | | 0.1 | 0.1 | 0.1 | 0.0 | 0.1 | 0.1 | 0.1 | 0.0 |
| **B'12** | 0.9 | | 1.0 | 0.7 | 0.0 | 0.0 | 0.3 | 0.1 | 0.3 | 0.3 |
| **B'13** | 0.0 | | 0.0 | 0.0 | 0.0 | 0.0 | 0.0 | 0.0 | 0.0 | 0.0 |
| **B'14** | 0.0 | | 0.0 | 0.1 | 0.0 | 0.0 | 0.1 | 0.0 | 0.1 | 0.0 |
| **B'15** | 2.3 | | 2.3 | 2.6 | 0.6 | 0.9 | 0.7 | 0.7 | 0.4 | 2.0 |
| **B'16** | 0.0 | | 0.0 | 0.0 | 0.0 | 0.0 | 0.0 | 0.0 | 0.0 | 0.0 |
| **B'17** | 0.0 | | 0.0 | 0.0 | 0.0 | 0.0 | 0.0 | 0.0 | 0.0 | 0.0 |
| **B'18** | 0.1 | | 0.2 | 0.2 | 0.2 | 0.1 | 0.0 | 0.1 | 0.1 | 0.0 |
| **B'19** | 0.0 | | 0.0 | 0.0 | 0.0 | 0.0 | 0.0 | 0.0 | 0.0 | 0.0 |
| **B'20** | 0.0 | | 0.0 | 0.0 | 0.0 | 0.0 | 0.0 | 0.0 | 0.0 | 0.0 |
| **B'21** | 0.0 | | 0.0 | 0.0 | 0.0 | 0.0 | 0.0 | 0.0 | 0.0 | 0.0 |
| **B'22** | 0.0 | | 0.0 | 0.0 | 0.0 | 0.0 | 0.0 | 0.0 | 0.0 | 0.0 |
| **B'23** | 0.0 | | 0.0 | 0.0 | 0.0 | 0.0 | 0.0 | 0.0 | 0.0 | 0.0 |
| **B'24** | 0.1 | | 0.3 | 0.0 | 0.2 | 0.0 | 0.0 | 0.1 | 0.2 | 0.0 |

**Supplementary Table 12.** PE population percentages changing temporally during the temperature shift from 60°C to 63°C. Samples were collected between 1200 and 1300 h.

| **% PE over time** | | | | | | |
| --- | --- | --- | --- | --- | --- | --- |
|  | **Day 0** | **Day 2** | **Day 4** | **Day 6** | **Day 2** | **Day 4** |
| **A1** | 4.3 | 24.5 | 36.0 | 16.8 | 23.4 | 18.2 |
| **A2** | 0.0 | 0.0 | 0.1 | 0.8 | 0.3 | 0.0 |
| **A3** | 0.1 | 0.1 | 0.9 | 0.3 | 0.5 | 0.1 |
| **A4** | 5.3 | 3.6 | 13.4 | 4.7 | 2.7 | 7.2 |
| **A5** | 3.0 | 3.3 | 20.5 | 4.1 | 4.4 | 3.2 |
| **A6** | 13.2 | 7.9 | 8.9 | 12.2 | 5.0 | 6.9 |
| **A7** | 0.0 | 0.3 | 3.4 | 0.3 | 1.0 | 0.3 |
| **A'8** | 0.0 | 0.0 | 0.0 | 0.1 | 0.0 | 0.0 |
| **A'9** | 0.0 | 0.3 | 0.0 | 0.0 | 0.3 | 0.3 |
| **A10** | 0.1 | 0.4 | 0.1 | 0.2 | 0.4 | 0.1 |
| **A11** | 0.2 | 0.0 | 1.0 | 0.3 | 0.0 | 0.8 |
| **A12** | 0.1 | 0.6 | 1.0 | 0.5 | 0.5 | 1.7 |
| **A13** | 0.0 | 0.0 | 0.0 | 0.2 | 1.0 | 0.2 |
| **A14** | 3.8 | 15.6 | 5.1 | 14.6 | 4.9 | 15.8 |
| **A15** | 0.1 | 0.2 | 0.4 | 0.2 | 0.2 | 1.0 |
| **A'16** | 0.0 | 0.2 | 0.0 | 0.2 | 0.0 | 0.0 |
| **A'17** | 0.0 | 0.0 | 0.0 | 0.0 | 0.0 | 0.0 |
| **A'18** | 0.0 | 0.0 | 1.3 | 0.1 | 0.1 | 0.1 |
| **A19** | 0.0 | 0.0 | 0.0 | 0.0 | 0.0 | 0.0 |
| **A20** | 0.0 | 0.0 | 0.0 | 0.2 | 0.0 | 0.0 |
| **A'21** | 0.0 | 0.0 | 0.3 | 0.0 | 0.2 | 0.0 |
| **A'22** | 0.0 | 0.0 | 0.1 | 0.0 | 0.3 | 0.2 |
| **B'1** | 0.4 | 0.0 | 0.0 | 0.0 | 0.2 | 0.0 |
| **B'2** | 10.2 | 1.7 | 0.0 | 2.2 | 4.1 | 2.9 |
| **B'3** | 0.2 | 0.2 | 0.2 | 0.3 | 0.2 | 0.3 |
| **B'4** | 0.0 | 0.0 | 0.0 | 0.0 | 0.1 | 0.0 |
| **B'5** | 0.0 | 0.9 | 0.3 | 0.6 | 1.4 | 1.3 |
| **B'6** | 0.0 | 0.0 | 0.0 | 0.0 | 0.1 | 0.0 |
| **B'7** | 0.4 | 0.0 | 0.6 | 0.2 | 0.5 | 0.1 |
| **B'8** | 8.7 | 7.4 | 1.2 | 7.2 | 4.1 | 4.7 |
| **B'9** | 23.7 | 9.7 | 3.6 | 17.5 | 13.3 | 13.4 |
| **B'10** | 0.2 | 0.2 | 0.0 | 0.2 | 0.3 | 0.0 |
| **B'11** | 0.5 | 0.5 | 0.0 | 0.4 | 0.2 | 0.4 |
| **B'12** | 3.8 | 0.4 | 0.3 | 1.2 | 2.8 | 2.2 |
| **B'13** | 0.1 | 0.0 | 0.0 | 0.0 | 0.0 | 0.1 |
| **B'14** | 0.0 | 0.0 | 0.1 | 0.1 | 0.5 | 0.0 |
| **B'15** | 0.4 | 8.2 | 1.1 | 2.8 | 12.6 | 6.3 |
| **B'16** | 0.0 | 0.0 | 0.0 | 0.0 | 0.0 | 0.0 |
| **B'17** | 0.5 | 0.6 | 0.0 | 0.1 | 0.3 | 0.1 |
| **B'18** | 0.7 | 0.1 | 0.0 | 0.3 | 0.5 | 0.9 |
| **B'19** | 0.1 | 0.0 | 0.0 | 0.0 | 0.0 | 0.0 |
| **B'20** | 0.0 | 0.3 | 0.6 | 0.0 | 0.0 | 0.0 |
| **B'21** | 0.0 | 0.0 | 0.0 | 0.0 | 0.0 | 0.0 |
| **B'22** | 0.0 | 0.2 | 0.0 | 0.1 | 0.1 | 0.1 |
| **B'23** | 0.1 | 0.0 | 0.0 | 0.1 | 0.0 | 0.0 |
| **B'24** | 0.1 | 0.1 | 0.2 | 0.2 | 0.3 | 0.1 |

**Supplementary Table 13. PE population percentages over time during the light alteration experiments conducted at 63°C. Samples were collected between 1200 and 1300 h.**

|  |  | **Time** | | | | | | | | |
| --- | --- | --- | --- | --- | --- | --- | --- | --- | --- | --- |
|  |  | **Light reduction** | | | | | |  | **UV removal** | |
|  |  | **Replicate sample 1** | | |  | **Replicate sample 2** | |  |  | |
| **PE** |  | **Day 0** | **Day 2** | **Day 4** |  | **Day 2** | **Day 4** |  | **Day 2** | **Day 4** |
| **A1** |  | 13.0 | 20.6 | 19.5 |  | 14.5 | 13.4 |  | 10.4 | 10.0 |
| **A2** |  | 8.4 | 5.5 | 3.8 |  | 5.8 | 5.2 |  | 3.6 | 4.1 |
| **A3** |  | 0.3 | 0.1 | 0.1 |  | 0.1 | 0.3 |  | 0.0 | 0.0 |
| **A4** |  | 8.2 | 3.2 | 2.5 |  | 1.7 | 0.3 |  | 2.2 | 1.2 |
| **A5** |  | 5.4 | 6.1 | 4.6 |  | 4.8 | 8.7 |  | 1.5 | 3.2 |
| **A6** |  | 24.2 | 11.6 | 9.3 |  | 13.5 | 11.5 |  | 7.9 | 11.5 |
| **A7** |  | 7.3 | 18.1 | 10.1 |  | 7.6 | 7.5 |  | 4.6 | 7.1 |
| **A'8** |  | 0.0 | 0.0 | 0.0 |  | 0.0 | 0.0 |  | 0.0 | 0.0 |
| **A'9** |  | 0.0 | 0.2 | 0.7 |  | 0.3 | 0.8 |  | 0.1 | 0.3 |
| **A10** |  | 1.2 | 1.6 | 0.3 |  | 4.0 | 1.0 |  | 0.3 | 1.0 |
| **A11** |  | 2.5 | 1.3 | 2.4 |  | 0.5 | 0.8 |  | 1.9 | 0.3 |
| **A12** |  | 5.9 | 16.2 | 10.2 |  | 9.1 | 20.3 |  | 6.3 | 6.3 |
| **A13** |  | 0.1 | 0.2 | 0.1 |  | 0.0 | 0.0 |  | 0.2 | 0.2 |
| **A14** |  | 5.2 | 1.8 | 1.8 |  | 3.5 | 7.6 |  | 2.1 | 0.9 |
| **A15** |  | 0.1 | 0.0 | 0.1 |  | 0.2 | 0.5 |  | 0.0 | 0.0 |
| **A'16** |  | 0.0 | 0.0 | 0.2 |  | 0.0 | 0.0 |  | 0.0 | 0.0 |
| **A'17** |  | 0.0 | 0.1 | 0.1 |  | 0.1 | 0.1 |  | 0.0 | 0.0 |
| **A'18** |  | 2.4 | 5.3 | 2.8 |  | 2.5 | 3.2 |  | 0.6 | 1.6 |
| **A19** |  | 0.0 | 0.0 | 0.0 |  | 0.0 | 0.0 |  | 0.0 | 0.0 |
| **A20** |  | 0.0 | 0.0 | 0.1 |  | 0.0 | 0.0 |  | 0.0 | 0.0 |
| **A'21** |  | 0.0 | 0.1 | 0.4 |  | 0.0 | 0.3 |  | 0.1 | 0.0 |
| **A'22** |  | 2.0 | 1.4 | 0.7 |  | 0.7 | 0.2 |  | 0.3 | 0.6 |
| **B'1** |  | 1.7 | 0.0 | 1.1 |  | 0.8 | 0.1 |  | 1.1 | 2.0 |
| **B'2** |  | 0.0 | 0.0 | 0.1 |  | 0.0 | 0.1 |  | 0.1 | 0.3 |
| **B'3** |  | 0.0 | 0.0 | 0.0 |  | 0.0 | 0.0 |  | 0.1 | 0.0 |
| **B'4** |  | 0.0 | 0.0 | 0.0 |  | 0.0 | 0.0 |  | 0.0 | 0.0 |
| **B'5** |  | 0.2 | 0.0 | 0.0 |  | 0.3 | 0.3 |  | 0.4 | 0.2 |
| **B'6** |  | 0.0 | 0.0 | 0.0 |  | 0.0 | 0.0 |  | 0.0 | 0.0 |
| **B'7** |  | 0.2 | 0.1 | 0.4 |  | 0.4 | 0.2 |  | 0.6 | 0.4 |
| **B'8** |  | 0.4 | 0.0 | 0.5 |  | 0.4 | 1.0 |  | 0.6 | 0.4 |
| **B'9** |  | 4.5 | 2.0 | 5.1 |  | 2.9 | 3.1 |  | 17.1 | 14.8 |
| **B'10** |  | 0.1 | 0.0 | 0.0 |  | 0.4 | 0.2 |  | 0.6 | 0.4 |
| **B'11** |  | 0.1 | 0.0 | 0.3 |  | 0.3 | 0.2 |  | 0.4 | 0.0 |
| **B'12** |  | 0.8 | 0.1 | 1.2 |  | 1.0 | 0.0 |  | 1.1 | 1.3 |
| **B'13** |  | 0.0 | 0.0 | 0.0 |  | 0.0 | 0.0 |  | 0.0 | 0.0 |
| **B'14** |  | 1.5 | 1.7 | 5.7 |  | 3.1 | 2.6 |  | 5.9 | 6.1 |
| **B'15** |  | 5.6 | 1.9 | 10.4 |  | 17.1 | 4.0 |  | 16.2 | 14.3 |
| **B'16** |  | 0.0 | 0.0 | 0.0 |  | 0.0 | 0.0 |  | 0.0 | 0.0 |
| **B'17** |  | 0.0 | 0.0 | 0.0 |  | 0.0 | 0.0 |  | 0.0 | 0.0 |
| **B'18** |  | 0.0 | 0.0 | 0.0 |  | 0.0 | 0.0 |  | 0.0 | 0.0 |
| **B'19** |  | 0.0 | 0.0 | 0.0 |  | 0.0 | 0.0 |  | 0.0 | 0.0 |
| **B'20** |  | 0.1 | 0.7 | 1.0 |  | 3.6 | 0.6 |  | 0.3 | 0.1 |
| **B'21** |  | 0.0 | 0.0 | 0.4 |  | 0.0 | 0.0 |  | 0.0 | 0.0 |
| **B'22** |  | 0.0 | 0.0 | 0.0 |  | 0.0 | 0.0 |  | 0.0 | 0.0 |
| **B'23** |  | 0.0 | 0.0 | 0.0 |  | 0.0 | 0.0 |  | 0.0 | 0.0 |
| **B'24** |  | 0.4 | 0.0 | 0.1 |  | 0.0 | 0.0 |  | 0.4 | 0.2 |


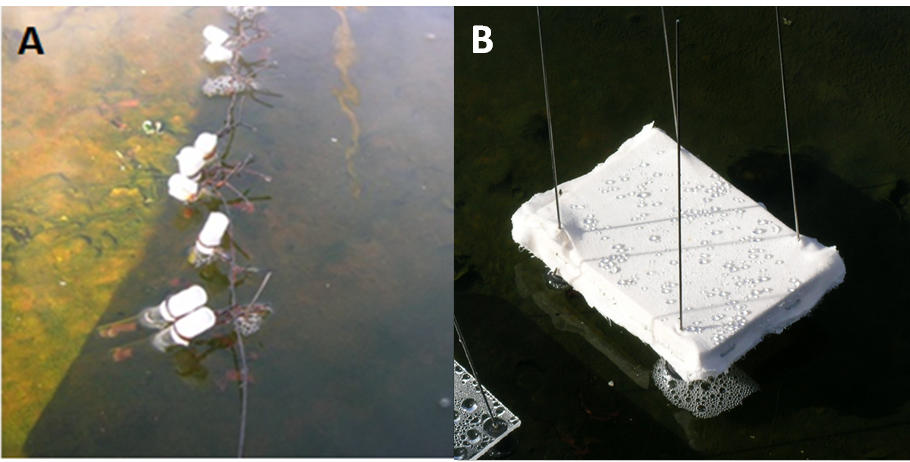


**Supplementary Figure 1.** Photographs of the temperature shift and light reduction experiments. (A) 3 ml vials suspended by an aluminum wire, used to suspend samples in spring water for temperature-shift experiments. (B) Muslin light-reducing cover used in light alteration experiment conducted at 63°C.


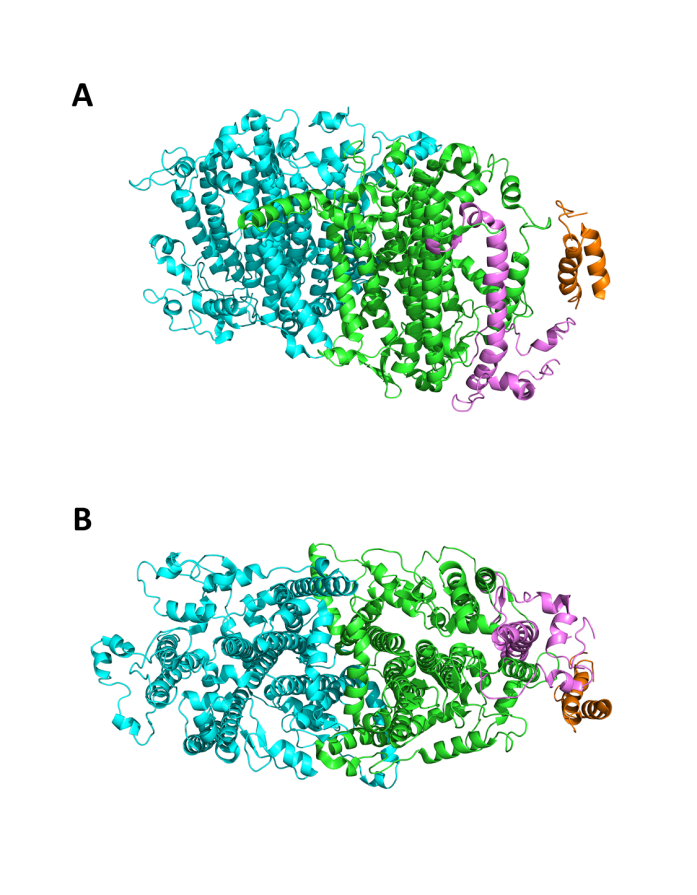


**Supplementary Figure 2.** Side view (A) and lumenal side view (B) of the X-ray structure of PsaA (green), PsaB (blue), and PsaK (orange) subunits of the Photosystem I complex of *Synechococcus elongatus* (PDB: IJB0; Jordan et al., 2001). Amino acids encoded by the PCR-amplified portion of *psaA* (amino acids 178 to 285) of PsaA are shown in pink. These correspond to the last three residues of transmembrane helix 2, the short stromal side loop connecting helices 2 and 3, transmembrane helix 3, and the lumenal side loop that connects helices 3 and 4. Note that three residues, Gly-Val-Ile (residues 272 to 274; Gly-Val-Val in *Synechococcus* PsaA) are unstructured in the crystal structure and thus are not shown in the figure; this accounts for the short gap in the loop near PsaK. Much of this region of PsaA is surface-exposed, and other than limited interactions with PsaK, this region does not interact with other subunits of PS I or with electron transport partners such as cytochrome *c*_6_ or ferredoxin. Likewise, this region is distant from the electron transfer cofactors of PS I. This region binds two β-carotene molecules and contains seven conserved histidine residues that ligate seven chlorophyll *a* molecules.


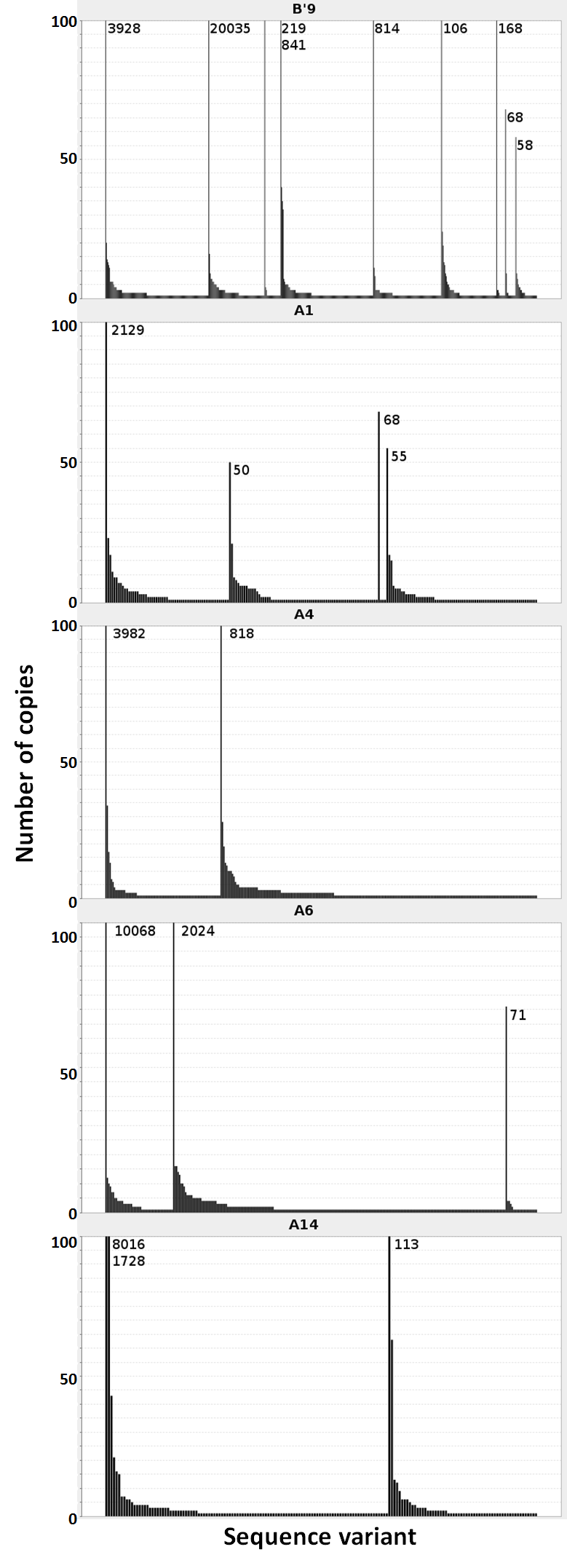


**Supplementary Figure 3.** Frequency plots of *psaA* variants predicted to belong to the same putative ecotype populations, which are distributed progressively from top to bottom in the 60-63°C Mushroom Spring microbial mat upper-green layer. Each position along the x-axis represents a unique sequence. High-frequency sequence variants are capped at 100 occurrences to enhance visualization of low-frequency sequence variants. The actual number of occurrences are presented next to high-frequency sequence. Less-abundant variants are shown to the right of each high-frequency sequence variant with which they are associated.


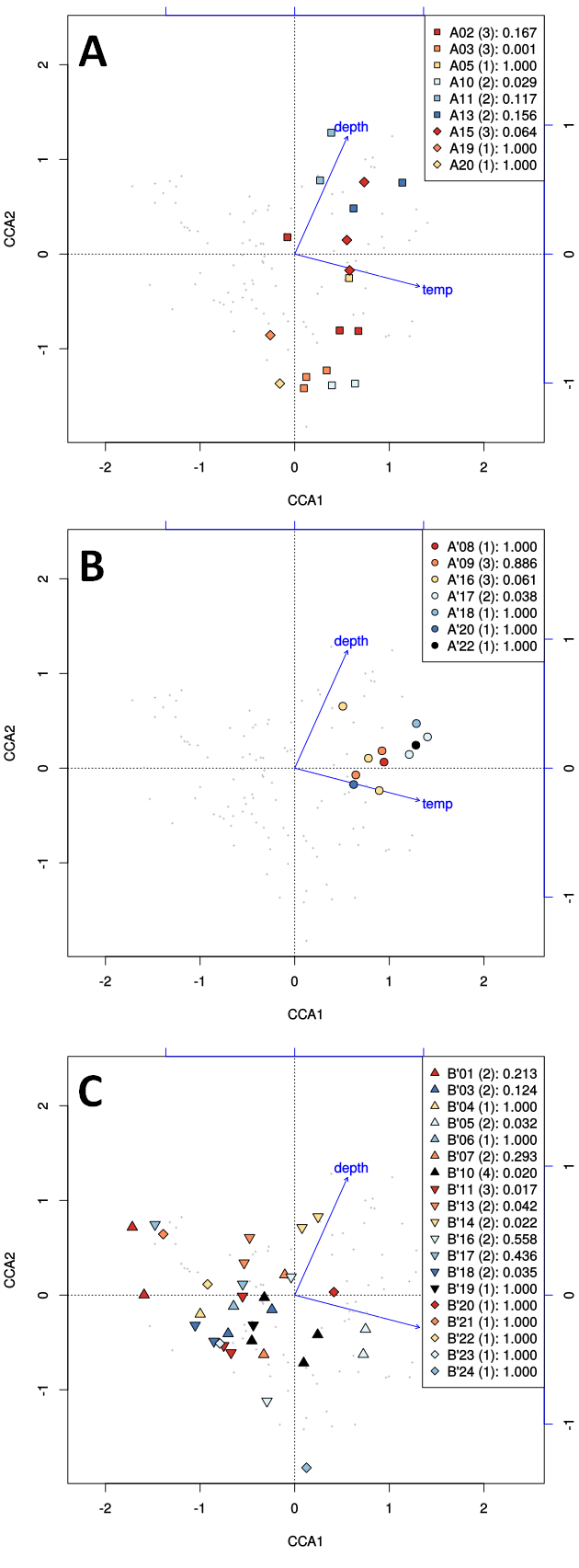


**Supplementary Figure 4.** Canonical correspondence analyses of high-frequency *psaA* sequence variants predicted by Ecotype Simulation to belong to the same non-predominant targeted putative ecotype (PE) for A′-like (A), A-like (B) and B′-like (C) *Synechococcus* putative ecotypes. Small grey data points represent high-frequency sequence variants detected in analysis of six vertical profiles in replicate samples from 60, 63 and 65°C. High-frequency sequences that represent PEs are color-highlighted as indicated by the inset together with the number of variants and the p-value associated with the cluster being different from random.


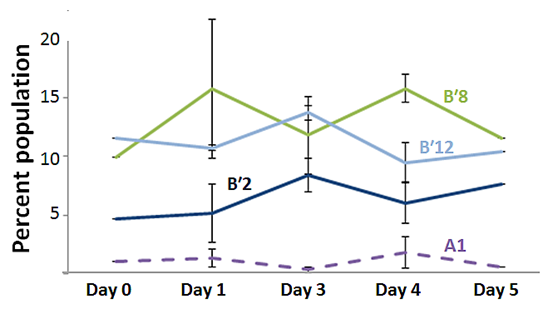


**Supplemental Figure 5.** Percent population of putative ecotypes in light alteration control samples over a five day period. Experiments were conducted at ~60°C in 1996 by David Ward. Bars indicate the range between replicate samples.
